# ﻿Taxonomic revision of the southern African species of the genus *Cynoglossum* L. (Boraginaceae)

**DOI:** 10.3897/phytokeys.193.72270

**Published:** 2022-03-15

**Authors:** Lydia K. Madika, Annah Ntsamaeeng Moteetee

**Affiliations:** 1 Department of Botany & Plant Biotechnology, Faculty of Science, University of Johannesburg, PO Box 524 Auckland Park 2006, Johannesburg, South Africa University of Johannesburg Johannesburg South Africa

**Keywords:** Boraginaceae, *Cynoglossum*, Southern Africa, taxonomic revision

## Abstract

The aim of the study is to provide a revision of the genus *Cynoglossum* in southern Africa. The genus is taxonomically problematic within the family Boraginaceae, due to the morphological similarities it shares with other closely related genera in the family. *Cynoglossum* plants are low-growing biennial, perennial, or rarely annual herbs which are recognizable by their hairy stems and leaves, the latter are usually basal and long petiolate. Based on the latest checklist, a total of eight species of this genus are listed for the study region: *C.alticola*, *C.amabile*, *C.austroafricanum*, *C.geometricum*, *C.hispidum*, *C.lanceolatum*, *C.obtusicalyx* (endemic to South Africa), and *C.spelaeum*. The occurrence of *C.amabile* in the region, however, requires further investigation since the only existing specimen was collected within a protected area in the KwaZulu-Natal province. Two specimens collected in the Doornpoort area in Pretoria, Gauteng province, assigned to this species appear to have been misidentified. Diagnostic characters are described, correct nomenclature, synonyms, typification, distribution maps, as well as the key for identifying the studied species, are provided.

## ﻿Introduction

The type genus *Cynoglossum* L. of the tribe Cynoglosseae belongs to the angiosperm family Boraginaceae (forget-me-not or borage family). Members of this genus have a worldwide distribution with many species occupying the temperate as well as the tropical regions of the Old and New Worlds. This genus comprises about 80–90 (Johnston 1924), to ca. 100 (Weigend et al. 2013), or as many as 200 species (Chacón et al. 2016). The highest diversity of species occurs in the Mediterranean region, harbouring about 20 species (Selvi and Sutorý 2012), and only a few species are introduced in Australia and one in North America (i.e., *C.creticum*) (Selvi et al. 2011). The name *Cynoglossum* is derived from the Greek words ‘*cynos*’ (of a dog) and ‘*glossa*’ (tongue), depicting the texture and shape of the leaves in species of the genus (Retief 2005), hence the common name ‘hound’s tongue’. This genus was established by Linnaeus (1753) to accommodate six species that he described at the time, namely, *Cynoglossumofficinale* L., *C.virginianum* L., *C.cheirifolium* L., *C.apenninum* L., *C.linifolium* L., and *C.omphaloides* L. The present state of knowledge has renamed most of these Linnaeus names to the following accepted names, i.e., *C.virginianum* L. (=*Andersonglossumvirginianum* (L.) J.I. Cohen), *C.cheirifolium* L. (=*Pardoglossumcheirifolium* (L.) Barbier & Mathez), *C.apenninum* L. (=*Solenanthusapenninum* (L.) Fisch. & C.A. Mey.), *C.linifolium* L. (=*Iberodeslinifolium* (L.) Serrano, R. Carbajal & S.Ortiz), and *C.omphaloides* L.(=*Omphalodesverna* Moench).

The genus is complex and has been noted by several botanists such as Miller (2005), Sutorý (2010), Selvi and Sutorý (2012), to be taxonomically challenging within the family due to the limited morphological variation between the species, which has rendered identification difficult. Another unresolved problem concerns the species relationships in *Cynoglossum*. Morphological characters that were traditionally used to delineate genera proved to be insufficient, especially in defining monophyletic groups within *Cynoglossum* as stated by Hilger et al. (2015). Recent phylogenetic studies by Cohen (2011, 2013, 2015), Selvi and Sutorý (2012), and Weigend et al. (2013), revealed that *Cynoglossum* is paraphyletic with respect to *Cynoglossopsis* Brand, *Lindelofia* Lehm., *Paracaryum* Boiss., *Pardoglossum* Barbier & Mathez, *Rindera* Pall., *Solenanthus* Ledeb., and *Trachelanthus* Kunze, suggesting the need for generic realignment. As a contribution to the systematics of the family, Johnston (1924) grouped half a dozen of genera under a broad concept of *Cynoglossum* based on the character of corolla shape and degree of anther exertion. From this grouping, the genus *Rindera* (=*Mattia*) and *Paracaryum* (=*Mattiastrum*) were still recognised as distinct with the only diagnostic feature being the fruit ornamentation. *Cynoglossum* portraying zoochory adapted glochidiate dispersal units, *Rindera* with the broadly winged diaspores for wind dispersal, and *Paracaryum* exhibiting the combinatory features (Greuter 1981). This unresolved relationship of taxa based on morphological traits led to authors such as Greuter and Burdert’s (Greuter 1981) request a broader conception of this genus, where *Cynoglossumsensu lato* forms a single, large genus.

An article by Von Staden et al. (2013), mentioned that taxonomically problematic genera are most likely to result in poor collection and misidentification. The latter case has become a reality for the southern African species of *Cynoglossum*, whereby members of this genus are also confused with species from other closely related genera such as *Lithospermum* L. and *Myosotis* L., and members of the tribe Eritrichieae Gϋrke. *Lithospermum* and *Myosotis* members are characterized by ovoid nutlets with broad basal attachment scars and flat gynobases, while in *Cynoglossum* the nutlets are depressed-ovoid or subcircular with scars restricted to the apical half of the ventral surface and have narrowly conical gynobases (Weigend et al. 2013; Otero et al. 2014). Eritrichieae are differentiated from *Cynoglossum* based on the shape of the gynobase, i.e., narrow pyramidal to subulate gynobase and mostly small nutlets, as opposed to broadly pyramidal gynobase and mostly larger nutlets for *Cynoglossum* (Hasenstab-Lehman and Simpson 2012; Weigend et al. 2013). According to Al-Shehbaz (1991), *Cynoglossum* can also be distinguished from the closely related genera *Solenanthus* Ledeb. and *Trachelanthus* Kunze in having included, instead of exerted, stamens and lastly, from *Pardoglossum* Barbier & Mathez in having slim, glabrous glochids on the nutlets, instead of swollen glochids that are densely packed with minute papillae. The fruits in members of this genus and the floral morphological characters, are normally considered to be of diagnostic importance. The nutlets of tropical *Cynoglossum* are generally smaller than those of species found in the temperate regions (Al-Shehbaz 1991).

In the southern African region, this genus has not yet been taxonomically revised since the last treatment by Wright (1904), where he recognised two species, *C.enerve* Turcz. (now *C.hispidum* Thunb.) and *C.micranthum* Desf. He cited two species, namely, *C.leptostachyum* DC. and *C.hispidum* Thunb. as being imperfectly known. Hilliard and Burtt (1986) recorded the occurrence of Cynoglossumcoeruleumsubsp.johnstoniivar.mannii (Baker and C.H. Wright) Verdc. [recognised in southern Africa as *C.geometricum* Baker & C.H. Wright] for the first time in this region, while Retief and Van Wyk (1996) recorded *C.obtusicalyx* Retief & A.E. van Wyk, the only species endemic to South Africa. While a comprehensive revision is still lacking for southern African species, revisionary work has been undertaken mainly at regional or country level, for example East Africa (Verdcourt 1991), China (Shu 1995), Comoro Islands and Madagascar (Miller 2005), Italy (Selvi and Sutorý 2012), Nepal (Kӧnig et al. 2015), and Taiwan (Hsiao and Liu 1998). According to the recent checklist by Germishuizen and Meyer (2003), there are eight species of *Cynoglossum* occurring in southern Africa, namely *C.alticola* Hilliard & B.L. Burtt, *C.amabile* Stapf. & J.R. Drumm, *C.austroafricanum* Hilliard & B.L. Burtt, *C.geometricum* Baker & C.H. Wright, *C.hispidum* Thunb., *C.lanceolatum* Forssk., *C.obtusicalyx* Retief & A.E. van Wyk (endemic to South Africa), and *C.spelaeum* Hilliard & B.L. Burtt. This paper aims to provide a taxonomic revision of the southern African species, to provide a diagnostic key, as well as to lay out their distribution maps in the region.

## ﻿Materials and methods

A total of 316 specimens loaned from the following herbaria: PRE, GRA, NH, NU, NBG, and SAM (herbarium acronyms following Thiers 2019), were examined for distribution and morphological data. The type specimens of relevant species were studied online from the JSTOR website (https://plants.jstor.org/). Voucher specimens and plant material were collected during several field trips conducted throughout South Africa and Lesotho between October 2018 and December 2019. Specimens collected were deposited in JRAU. The system by Edwards and Leistner (1971) was used for specimen citation under the section ‘additional specimen examined’.

Data on vegetative morphology was obtained by analysing all the specimens provided per species. Inflorescence structures were studied from the freshly collected samples, herbarium samples, as well as from the original author’s descriptions (in a case where all the specimens did not contain any inflorescence to study). Hand drawings representing both the vegetative and reproductive characters were made for all the species (except for *C.amabile*). The trichomes from the leaf samples and mature nutlets, from as many specimens as possible, were examined per species, except for *C.amabile*, where there was only one specimen available, and *C.obtusicalyx*, where three specimens were available. Original descriptions from JSTOR.org were also included in developing the diagnostic key. The specimens were studied using either the TESCAN VEGA3 scanning electron microscope (SEM) or the Phenom Desktop SEM. For TESCAN VEGA3 SEM analysis, samples were immersed in a mixture of 95% ethanol and isoamyl acetate (1:1) for 10 minutes and in pure isoamyl acetate for 15 minutes. After removing isoamyl acetate, the samples were placed on a holder for critical point drying for an hour. Then the dried samples were directly mounted on aluminium stubs and sputter-coated with a thin layer of gold before viewing under microscope. This was done to prevent charging of specimens due to accumulation of static electric field, and to increase the number of secondary electrons that can be detected from the surface of the specimen. For the Phenom Desktop SEM analysis, samples were only air-dried for an hour and were directly mounted on the aluminium stubs and viewed under microscope. Maps were plotted using the program CorelDraw Graphics Suite X7 (http://coreldraw.com).

## ﻿Morphological characters of *Cynoglossum* species in southern Africa

### ﻿Vegetative morphology

**Figure 1.**
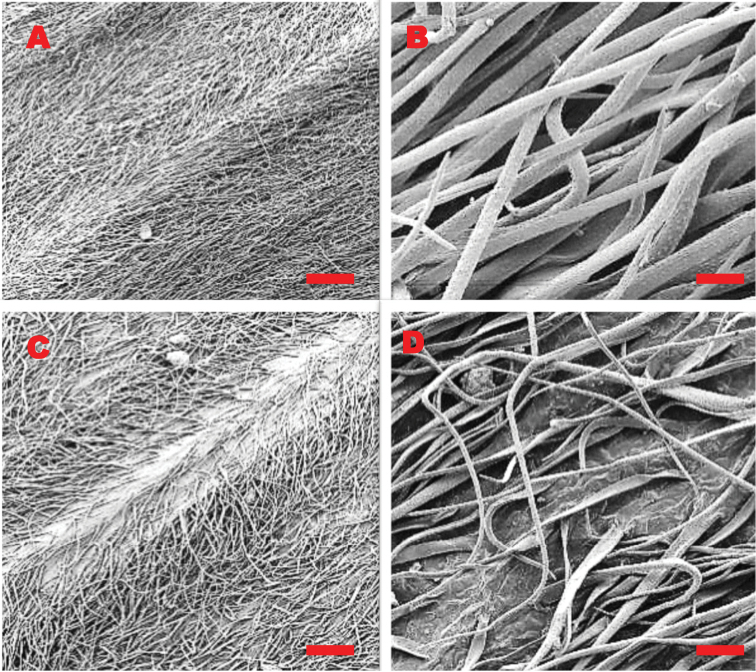
SEM micrographs of the adaxial leaf surfaces and the midrib of (**A, B**) *C.alticola* (**C, D**) *C.obtusicalyx***B** close-up of *C.alticola* displaying cylindrical trichomes with pointed end **D** close-up of *C.obtusicalyx* displaying flat trichomes with blunt tip. Vouchers: **A, B** from *L.C.C. Liebenberg 5789* (PRE) **C, D** from *J.P.H. Acocks 8509* (PRE). SEM images scale bars: 10 mm (**A**); 50 µm (**B**); 1 mm (**C**); 100 µm (**D**).

Members of this genus are either perennial, biennial, or rarely annual herbs which are recognizable by their hairy stems and leaves. Roots are thickened cream white taproots with small lateral roots. The stems are erect, hollow, simple at the base, and usually branched above. The basal leaves are deciduous, long petiolate, lanceolate-obtuse shaped, cross-venulate, with smooth margins, and are clustered at the lower parts of the stem forming a rosette, covered with simple trichomes on both the adaxial and abaxial surface. The stem leaves are alternate, sessile, or petiolate, lanceolate-obtuse shaped, with smooth margins. Trichomes on the leaves sometimes have a pustulate base typical of Boraginaceae members (*C.austroafricanum*, Cynoglossumcoeruleumsubsp.johnstoniivar.mannii and *C.lanceolatum*). Vegetative morphology is of limited diagnostic value in distinguishing between southern African species, however a closer look at the trichomes has shown that they may be used to distinguish between similar species. For example, both *C.alticola* and *C.obtusicalyx* have a cluster of soft, woolly trichomes which differ in shape (cylindrical with a pointed tip in *C.alticola*, vs. flat surface and spherical with a blunt tip in *C.obtusicalyx)* and density (denser in *C.alticola* than in *C.obtusicalyx*) as observed in Figure 1.

### ﻿Reproductive morphology

**Figure 2.**
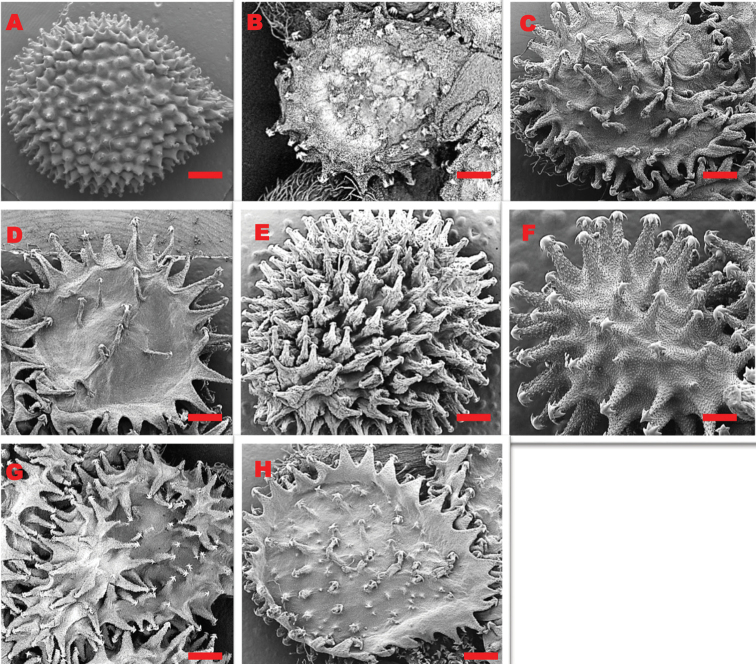
SEM micrographs of the adaxial surface of the nutlets of eight listed species of *Cynoglossum***A***C.alticola***B***C.amabile***C***C.austroafricanum***D***C.coeruleumvar.mannii***E***C.hispidum***F***C.lanceolatum***G***C.obtusicalyx***H***C.spelaeum*. Voucher specimens: A from *L.C.C. Liebenberg 5789* (PRE) **B** from *J. Stewart 2021* (NU) **C** from *O.M. Hilliard* and *B.L. Burtt 11803* (PRE) **D** from *T.B. Sikhakhane 440* (NH) **E** from *S.P. Bester 12958* (PRE) **F** from *S.P. Bester 4653* (PRE) **G** from *J.P.H. Acocks 8509* (PRE) **H** from *A. Nicholas* and *B. Isaacs 1965* (PRE). SEM images scale bars: 1 mm (**A, C-E, G-H**); 2 mm (**B**); 500 µm (**F**).

The inflorescence is a cyme which is often dichotomously branched with spreading panicles. Flowers are either pedicelled or subsessile, with five parted corollas; corolla white with a blue throat, blue, violet, or magenta (*C.hispidum*), or rarely white (*C.spelaeum*). The stamens are included and arise from the base of the tube, they have short filaments and elliptic to oblong shaped anthers. The style is short and relatively thick, with a capitate stigma. The fruit is a schizocarp of four nutlets attached apically to a narrowly conical gynobase. The nutlets are ovoid with a convex dorsal surface. At maturity, the nutlets produce glochidia, which are sharp hair-like spines or bristles tipped with barbs. The glochidia are either swollen at the base or not bulbous-based, they either cover the whole surface or are well spaced and vary in number. The structure and shape of glochidia display an important distinguishing character amongst the southern African species (Table 1), with each species portraying a unique character as can be seen in Figure 2.

**Table 1.** Distinguishing characters between the southern African *Cynoglossum* species.

Characters	*C.alticola*	*C.amabile*	*C.austroafricanum*	C.coeruleumsubsp. johnstoniivar.mannii	*C.hispidum*	*C.lanceolatum*	*C.obtusicalyx*	*C.spelaeum*
Floral colour	Deep blue	Bluish purple	Pale blue	White with pale blue throat	Magenta	White with pale blue throat	Pale blue	White
Nutlet shape and size	Convex, 6–9×5–6 mm	Ovoid to convex, 2–4×3–4 mm	Ovoid, 3.0–3.5×2.5–3.0 mm	Ovoid, 3–4×2.5–3.5 mm	Convex, 5–6×3–5 mm	Ovoid-convex, 3–4×2.5–3.5 mm	Ovoid-convex, 2–4×3–4 mm	Ovoid, ca. 4×5 mm
Nutlet ornamentation	Glochidia densely packed on nutlet	Marginal glochidia are more distinct than the central glochidia	Glochidia more spread towards the margins	Glochidia more marginal and on the median line	Glochidia covering the whole nutlet	Glochidia sparsely arranged on nutlet	densely echinulate with glochidia	glochidia more marginal and acentric, marginal glochidia are longer compared to the acentric glochidia

## ﻿Taxonomic treatment

*Cynoglossum* L., Sp. Pl. 1: 134 (1753) & Gen. Pl.: 5 (1754); Benth. and Hook. f., Gen. Pl. 2: 848 (1876); C.H. Wright in Fl. Cap. 4: 13 (1904); Baker and C.H. Wright in Fl. Trop. Afr. 4 (2): 51 (1905); Brand in Engl., Pflanzenr. 4: 252 (1921); Al-Shehbaz in J. Arnold Arbor.: 112 (1991); Selvi and Sutorý in Pl. Biosyst. 146 (2): 461–479 (2012); Hilger *et al*. in Biodivers. Data J. 3: e4831 (2015). Type species: *C.officinale* L.

*Paracynoglossum* Popov. Fl. URSS xix.: 717 (1953). Type species: *P.denticulatum* (DC.) Popov.

Perennial, biennial, or rarely annual herbs, often tall, up to 1,2 m in height, and slightly branched. Stems and leaves canescent. Indumentum white, simple or tubercled. Leaves alternate, lanceolate, obtuse or spathulate, entire; first year basal leaves form a rosette, lanceolate or obtuse, often long petiolate. Inflorescences usually elongate, rarely bracteate, sparingly branched or loosely paniculate. Flowers pedicelled or subsessile; blue or violet with distinct veins, rarely white. Calyx five-partite, scarcely enlarged in fruit, patent or reflexed. Corolla tube short, throat closed with obtuse or arched scales; five-lobed, imbricate, obtuse, patent. Stamens five, included in the corolla tube, included, with short filaments, anthers ovoid or shortly oblong, obtuse. Ovary with four distinct lobes from an almost flat receptacle; style short or rather long; stigma small, flat or sub capitate; ovules horizontal, fixed to the central angle of the cell. Nutlets four, depressed, scarcely produced at the apex, convex or flat on the dorsal side or surrounded by an elevated margin, glochidiate (hair-like spines or short prickles). Seeds straight or slightly curve

### ﻿Diagnostic key to the species

**Table:** 

1	Soft woolly hairs covering the entire plant; nutlets thickened; glochidia densely arranged on the nutlet	**1. *C.alticola***
–	Stiff bristle hairs covering the entire plant; nutlets slightly swollen; glochidia sparsely spaced on the nutlet	**2**
2	Nutlets 5 mm wide; glochidia thick at the base, fruit stalk up to 2 cm long	**5. *C.hispidum***
–	Nutlets less than 5 mm wide; glochidia uniformly shaped, fruit stalk up to 1 cm long	**3**
3	Inflorescences clustered at the apex; corolla bluish purple; glochidia dense at the margins and centre of the nutlet	**2. *C.amabile***
–	Inflorescences not clustered at the apex; corolla blue to white; glochidia dense at the margins and few at the centre of the nutlet	**4**
4	Spreading long trichomes covering the whole plant; corolla longer than 7 mm long	**7. *C.obtusicalyx***
–	Sparsely shorter trichomes covering the whole plant; corolla shorter than 4 mm long	**5**
5a	Corolla white with blue throat:	
6	Trichomes not thickened on both leaf surfaces; glochidia evenly distributed across the nutlet	**6. *C.lanceolatum***
–	Trichomes with pustulate base on the abaxial leaf surface; glochidia on the median line and centre of the nutlet	**4. Cynoglossumcoeruleumsubsp.johnstoniivar.mannii**
5b	Corolla uniformly coloured:	
7	Leaves brightly green coloured on both surfaces, lanceolate-obtuse shaped; corolla pale blue; length of glochidia uniform throughout the nutlet	**3. *C.austroafricanum***
–	Leaves grey green on the abaxial surface, dark green on the adaxial surface, spathulate-obtuse shaped; corolla white; marginal glochidia longer than acentric glochidia	**8. *C.spelaeum***

#### ﻿1. Cynoglossum alticola

Taxon classificationPlantaeBoraginalesBoraginaceae

Hilliard & B.L.Burtt, Notes Roy. Bot. Gard. Edinburgh 43(3): 343 (1986).

##### Type.

South Africa ♀♂ Eastern Cape, Barkly East District (3027): Ben McDhui (-DB), 5 Feb 1983, *O.M. Hilliard and B.L. Burtt 16468* (E-image!, holotype; NU-image!, isotype).

**Figure 3.**
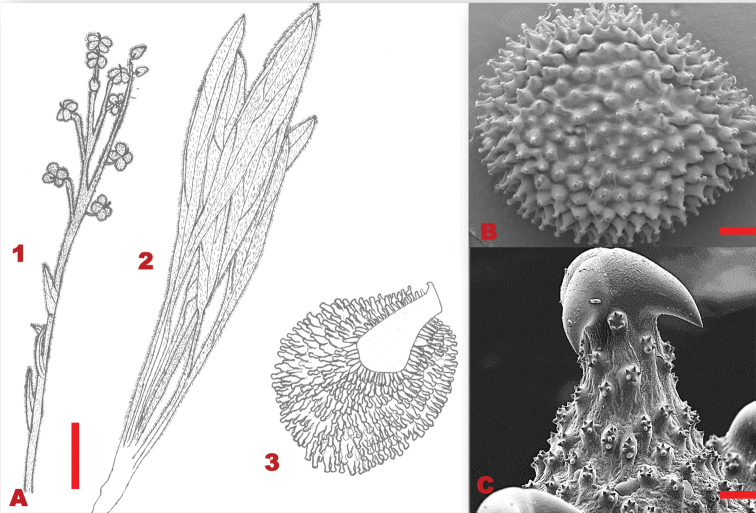
Vegetative and reproductive morphology of *Cynoglossumalticola***A** line drawings of: 1. a branch showing fruiting arrangement at the apex of the stem and stem leaves; 2. long petiolate basal leaves; 3. adaxial surface of nutlet **B**SEM micrograph showing trichomes on the leaves **C**SEM micrograph showing thick and shorter glochidia on the nutlet. Voucher: *L.C.C. Liebenberg 5789* (PRE). Drawing scale bar: 8 mm. SEM images scale bars: 50 µm (**B**); 50 µm (**C**).

Perennial herbs, 0.2–0.6 m in height. Basal leaves 76–270×8–18 mm, lanceolate, densely pubescent, persistent; margins entire. Stem leaves 35–120×5–10 mm, lanceolate, apex acute, base cuneate, margins entire, soft woolly hairs. Trichomes spread equally on both the adaxial and abaxial leaf surfaces, unicellular hair base, not bulbous on both leaf surfaces. Inflorescence racemose, clustered at the apex; pedicel 4–10 mm long, lengthening considerably in fruit. Calyx ca. 4 mm long, lobes elliptic-oblong, densely hairy on inner surface, apices obtuse. Corolla deep blue; lobe 4×3 mm diameter, oblong, round apex. Nutlets convex, 6–9×5–6 mm; glochidia short and thick at the base, densely packed on nutlet, tips multi-angular (Figure 3).

##### Phenology.

February to May.

##### Conservation status.

Least Concern (Raimondo et al. 2009).

##### Diagnostic characters.

*Cynoglossumalticola* can be distinguished by its thick, convex nutlets. Among the southern African species, it has a unique appearance due to the presence of woolly trichomes that cover the whole plant. Furthermore, it has larger nutlets (6–9×5–6 mm) than other species (less than 6×5 mm). According to Hilliard and Burtt (1986), this species is related to *C.alpinum* (Brand) B.L. Burtt from the highlands of Ethiopia, with which it shares nutlet shape and size, as well as the leaf texture and colour. The difference is observed on the fornices (small crests in the corolla tube of a plant), as in *C.alticola* they are broad and short while in *C.alpinum* they are long and narrow.

##### Distribution and habitat.

**Figure 4.**
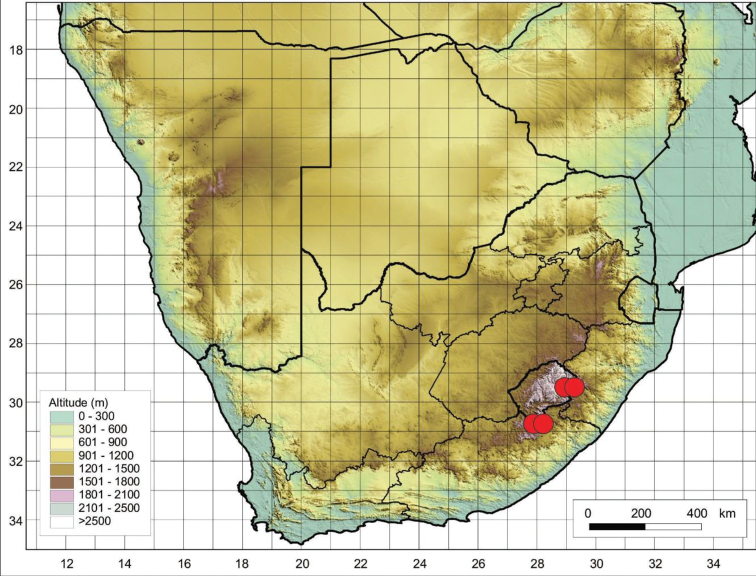
Distribution of *Cynoglossumalticola* based on the specimens examined.

The species is restricted to the Eastern Cape Province in South Africa and Lesotho (Figure 4), where it is found growing on mountainous terrain and on damp slopes near streams.

##### Additional specimens examined.

South Africa. Eastern Cape: 3028 (Matatiele): Drakensberg, near Barkly East (-CA), 19 Dec 1982, *P.B. Phillipson 705* (PRE); between Malpas and Nek (-CA), 13 Dec 1995, *T. Dold* and *M. Cocks 2058* (GRA).

Lesotho. 2929 (Underberg): Mokhotlong District (-AC), Jan 1953, *L.C.C. Liebenberg 5789* (PRE); 28 Feb 1947, *A. Jacot Guillarmod 997* (PRE).

#### ﻿2. Cynoglossum amabile

Taxon classificationPlantaeBoraginalesBoraginaceae

Stapf & J.R.Drumm. in Bull. Misc. Inform., Roy. Bot. Gard., Kew 6:202 (1906).

##### Type.

China ♀♂ Yunnan, Mengtsze, 1894, *W. Hancock 133* (K-image! [3 sheets], lectotype, designated by Verdcourt 1991).

**Figure 5.**
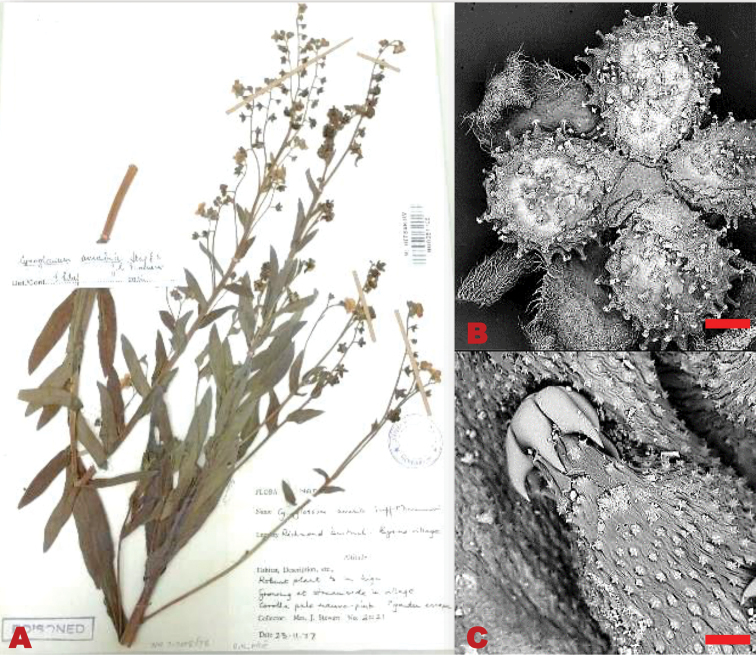
*Cynoglossumamabile*. SEM micrograph of the adaxial surface of **A** fruit nutlets **B** Glochidia. Voucher specimen: *J. Stewart 2021* (NU). SEM images scale bar: 2 mm (**A**); 100 µm (**B**).

Perennial herb, 0.6 m in height. Basal leaves 50–100×20–35 mm, lanceolate-elliptic shaped, softly hairy, deciduous, margins entire. Stem leaves 40–100×9–20 mm, lanceolate shaped, apex acute, base cuneate, entire margins, densely covered with white brittle hairs. Trichomes soft, upright, bulbous based. Inflorescence clustered at the apex, pedicels 5–8 mm long, lengthens considerably in fruit. Calyx ca. 3 mm long, lobes ovate, grey pubescent, apex subacute. Corolla bluish purple; lobe 7×9 mm diameter, segments round. Nutlets ovoid, 2–4×3–4 mm, convex shaped; glochidia short, thick, marginal glochidia are more distinct than the central glochidia (Figure 5).

##### Phenology.

October to November.

##### Conservation status.

Not evaluated (Raimondo et al. 2009).

##### Diagnostic characters.

Amongst the southern African species, *C.amabile* can be confused with *C.lanceolatum* due to their small-sized nutlets (between 2–4×2.5–4) and flowers. However, the two species are easily distinguished by their flower colour (*C.lanceolatum* has white corolla with blue throat, whereas *C.amabile* has bluish purple corolla). This species was also reported by Stapf and Drummond (1906) and Kӧnig et al. (2015) to be like *C.furcatum* Wallich (from Nepal, China, Bhutan, Vietnam, Thailand, Philippines, and India), based on flower and fruit size. The difference can be observed in the inflorescences, whereby *C.furcatum* is a much larger plant with inflorescences up to 1 m tall, and *C.amabile* is up to 0.6 m tall.

##### Distribution and habitat.

**Figure 6.**
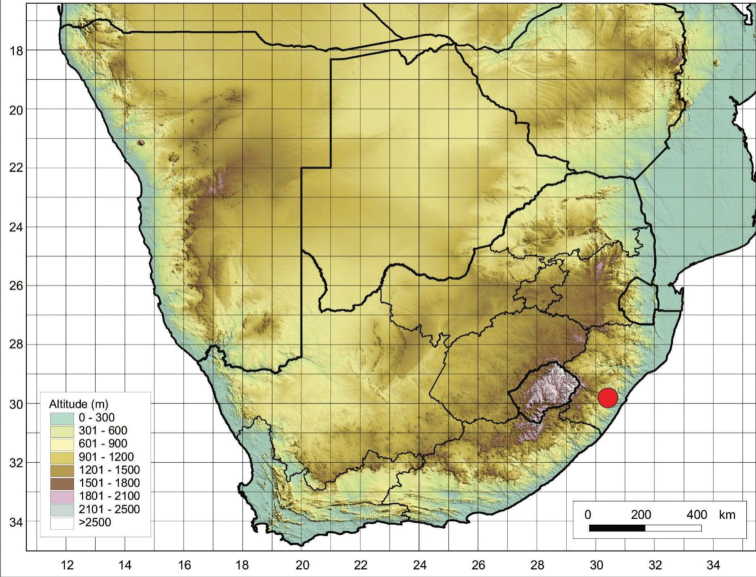
Recorded distribution of *Cynoglossumamabile* in southern Africa based on the specimens examined.

*Cynoglossumamabile* is widely distributed in southern China where it is usually grown for ornamental purposes and naturalised in many parts of the world (Xu et al. 2009). According to Germishuizen and Meyer (2003), this species is only found in KwaZulu-Natal Province (Figure 6), where it grows in open, disturbed sites, on gravel slopes and sandy, dry riverbanks.

##### Additional specimens examined.

South Africa. KwaZulu-Natal: 2930 (Pietermaritzburg): Richmond District, Byrne Village (-CD), 23 Nov 1977, *J. Stewart 2021* (NU).

##### Taxonomic notes.

*Cynoglossumamabile* has been described as a widespread species which grows in disturbed habitat and can be grown as an ornamental (Kӧnig et al. 2015). This species has only been collected once in South Africa by J. Stewart in 1977, since then there have been no later records or observations of this species in this region. Attempts to locate this species in the wild were futile. It is questionable whether this species occurs naturally in the southern African region since its single known locality is within a protected area in KwaZulu-Natal.

#### ﻿3. Cynoglossum austroafricanum

Taxon classificationPlantaeBoraginalesBoraginaceae

Weim. ex Hilliard & B.L. Burtt in Notes Roy. Bot. Gard., Edinburgh 43(3):347 (1986).

Cynoglossum austroafricanum Weim., Jacot Guillarmod, Fl. Lesotho: 233 (1971); Gibbs Russell *et al*., in Mem. Bot. Surv. S. Afr. 48:109 (1984), nom. nud.

##### Type.

South Africa ♀♂, KwaZulu-Natal, Underberg (2929): Cobham Forest Reserve, Sipongweni, c.6500ft (-CB), 21 Feb 1981, *O.M. Hilliard and B.L*. *Burtt 14072* (E-image! [2 sheets], holotype; K-image!; NU-image! [3 sheets], isotype).

**Figure 7.**
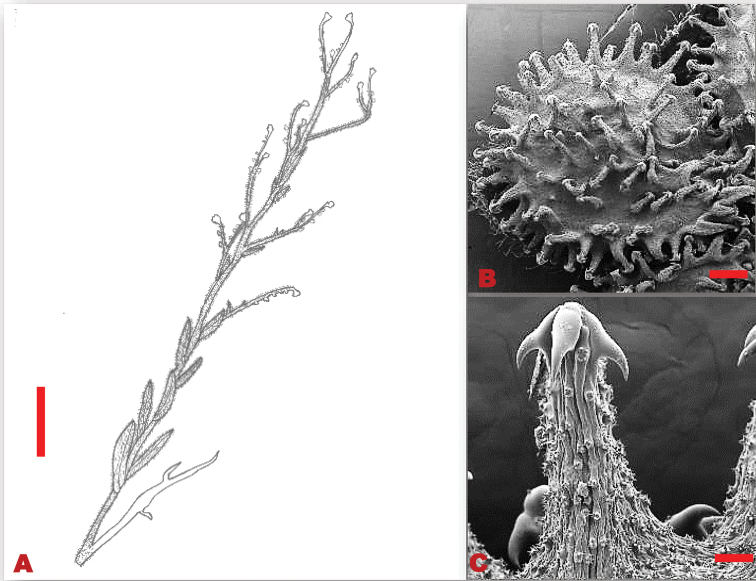
Vegetative and reproductive morphological features of *Cynoglossumaustroafricanum***A** line drawing of the branching pattern of the fruit stalk, and the alternating stem leaves **B**SEM micrograph of the adaxial surface of a fruit nutlet, with the arrangement of glochidia around the nutlet **C**SEM micrograph of the glochidia. Voucher specimen: *O.M. Hilliard* and *B.L. Burtt 11803* (PRE). Drawing scale bar: 7.5 mm. SEM scale bar: 1 mm (**B**); 100 µm (**C**).

Perennial or biennial herbs, 0.3–0.5 m in height. Basal leaves 100–190×15–30 mm, lanceolate-obtuse, softly hairy, persistent margins entire. Stem leaves 45–100×10–21 mm, narrowly lanceolate to linear lanceolate shaped, acute apex, cuneate base, margins undulate, covered with stiff hairs. Trichomes unicellular, with thick round base on the adaxial surface, simple on the abaxial surface. Inflorescences dichotomously branched, loose cymes at the apex, pedicels 4–9 mm long, and lengthens considerably in fruit. Calyx ca. 2–3 mm long, lobes obtuse, pubescent on the outer surface, glabrous on the inner surface, apex acute. Corolla pale blue; lobes 2.75–4.25 mm in diameter, cruciform, apex obtuse. Nutlets ovoid, 3.0–3.5×2.5–3.0 mm; glochidia more spread towards the margins, thin, tip multi-angular (Figure 7).

##### Phenology.

December to April.

##### Conservation status.

Least Concern (Raimondo et al. 2009).

##### Diagnostic characters.

Amongst the southern African species, *Cynoglossumaustroafricanum* can be confused with either *C.lanceolatum or C.coeruleumvar.mannii*. This species differs from the two by the colour of the corolla (white corolla with pale blue throat vs pale blue corolla throughout in *C.austroafricanum*). This latter observation was also noted by Hilliard and Burtt (1986).

##### Distribution and habitat.

**Figure 8.**
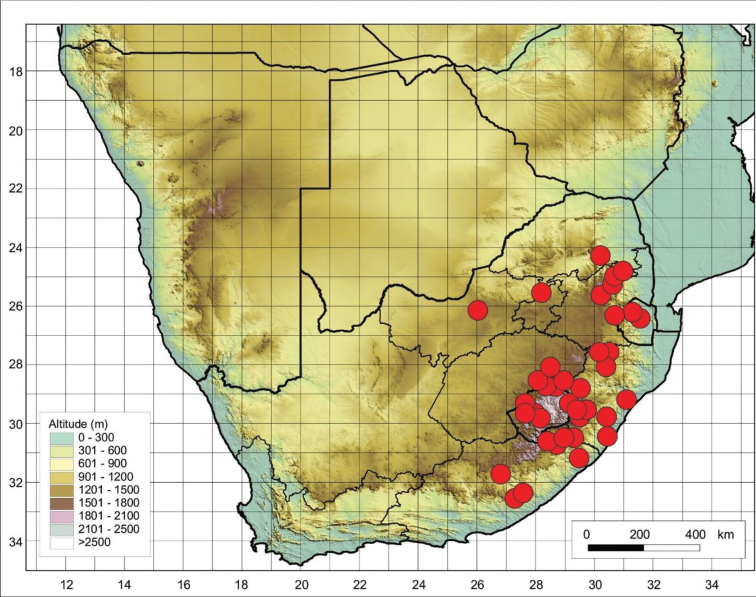
Known distribution of *Cynoglossumaustroafricanum* in southern Africa based on the specimens examined.

The species is distributed in South Africa (North-West, Gauteng, Mpumalanga, Free-State, KwaZulu-Natal, and Eastern Cape Provinces), eSwatini and Lesotho (Figure 8), where it occurs in shady, disturbed areas, and sandy, dry riverbanks.

##### Additional specimens examined.

South Africa. Limpopo: 2430 (Tzaneen): Lekgalameetse Nature Reserve (-AA), 7 Oct 1986, *N. Stalmans 1404* (PRE). North-West: 2626 (Lichtenburg): Lichtenburg (-AA), Feb 1918, *L. Kretzshmar 17065* (PRE). Gauteng: 2528 (Pretoria): Brooklyn (-CA), 1 Feb 1931, *A.O.D. Mogg 16451* (PRE). Mpumalanga: 2430 (Pilgrim’s rest): Pilgrim’s rest, next to the old railway line (DD), 31 Jan 2019, *A.N. Moteetee* and *L.K. Madika AL06* (JRAU). 2530 (Mashishing): Mashishing (-AB), Apr 1910, *M. Crosby 1989* (PRE); Mashishing District (-CA), 8 Feb 1904, *J. Burtt-Davy 1472* (PRE). 2630 (Carolina): Between Oshoek border post and Carolina (-BA), Jan 1906, *H. Bolus 12161* (PRE). Free State: 2828 (Bethlehem): Golden Gate (-AB), 22 Jan 1951, *A. Wiezer 22485* (NBG). (Fouriesburg): Bethlehem (-CA), 8 Jan 1918, *G. Potts 3246* (PRE); Witsieshoek (-DB), 28 Feb 1975, *O.M. Hilliard* and *B.L. Burtt 8665* (NU). Kwazulu-Natal: 2730 (Vryheid): Oshoek District, Wakkerstroom (-AC), 18 Jan 1961, *N.J. Devenish 480* (PRE); Amajoba District Municipality area, Luiperdkloof farm, Natural Heritage site no.47 (-AD), 25 Jan 2011, *A.M. Ngwenya 3601* (NH). 2829 (Harrismith): Cathedral Peak (-CC), 18 Feb 1983, *O.M. Hilliard* and *B.L. Burtt 16298* (NU); 12 Jan 1984, *J. Scott 76* (NH). 2830 (Dundee): Hattingspruit Station (-AA), Dec 1929, *D. Johnston 268* (NU). 2929 (Underberg): Mpendhle District, upper Loteni Valley (-AD), 5 Feb 1985, *O.M. Hilliard* and *B.L. Burtt 18103* (NU, PRE); Loteni Nature Reserve (-BC), 24 Dec 1978, *O.M. Hilliard* and *B.L. Burtt 11803* (PRE); Polela River (-CB), 21 Apr 1973, *M.A. Rennie 379* (NU); 23 Mar 1977, *O.M. Hilliard* and *B.L. Burtt 9793* (NU); 17 Feb 1982, *O.M. Hilliard* and *B.L. Burtt 15520* (NU). 2930 (Pietermaritzburg): Gate farm Keerom-Cottingham (-CC), 23 Mar 1969, *R.G. Strey 8420* (NH). 2931 (Stanger): Tugela valley (-AA), 14 Feb 1926, *Bayer 47* (NU). 3029 (Kokstad): Hillside (-CA), Jan 1956, *P. Thompson 2* (NU); on banks of streams near Kokstad (-CB), Dec 1883, *W. Tyson 1839* (NBG), 17 Jan 1957, *L.E. Taylor 5473* (NBG). 3030 (Dumisa): Port Shepstone (-AD), 22 Oct 1997, *J. Arkell 353* (NH). Eastern Cape: 3128 (Umtata): Transkei, on the summit of Baziya Mt. (-AD), 23 February 1988, *T. Strever 917* (PRE); Walter Sisulu University, Area 3: East of In-Service centre (-DB), 14 Feb 2001, *N. Nombekela 102* (NH). 3126 (Queenstown): Hangklip (-DD), Feb 1960, *H. Koepowitz 13147* (GRA). 3129 (Port St. John’s): Ntabankulu Mountain, Gome Forest Station, Tabankulu (-AB), 11 Nov 1996, *T. Dold*, *E. Cloete* and *R. White 2940* (GRA). 3227 (Stutterheim): Fort Cunynghame Station (-AD), Nov 1894, *T.R. Sim 1860* (NU); no date 1897, *T.R. Sim 20420* (NU).

eSwatini. 2631 (Mbabane): Forbes Reef (-AA), 14 Apr 1960, *R.H. Compton 30035* (NBG); Mbabane (-AC), 17 Jan 1951, *A. Wuze 22393* (NBG).

Lesotho. 2828 (Bethlehem): Leribe District, LHDA Phase 1A (-AD), 11 Jan 1996, *P.B. Phillipson*, *C. Mokuku*, *R. Judd*, and *C. Hobson 4473* (GRA); Leribe (-AD), no date, *M. Dieterlen 70* (NBG; NH). 2927 (Maseru): Thaba Bosiu (-BC), 1 Mar 1978, *M. Schmitz 8205* (PRE); Mahlatsa (-BB), 18 Jan 1941, *A. JacotGuillarmod 51* (GRA); Sefikeng Ha Fako (-BD), Oct 2018, *A.N. Moteetee 56* (JRAU); 30 Dec 2018, *A.N. Moteetee 59* (JRAU). 2929 (Underberg): Mokhotlong (-AC), Mar 1949, *A. Jacot Guillarmod 1072* (PRE), 25 Feb 1949, *W.J. Barker 21515* (NBG).

No locality details: 27 Nov 1888, *H. Medley 4576* (NH); Dec 1946, *E. Meston 50* (NU).

##### Taxonomic notes.

According to Hilliard and Burtt (1986), this species was described by Dr. H. Weimarck who did not select a type specimen; therefore, in typifying it they retained the specific epithet.

#### ﻿4. Cynoglossum coeruleum subsp. johnstonii var. mannii

Taxon classificationPlantaeBoraginalesBoraginaceae

(Baker & C.H. Wright) Verdc. in Fl.Trop. E. Africa, Boraginac.: 110 (1991).

Cynoglossum mannii Baker & C.H.Wright, in Oliver et al., Fl. Trop. Afr., 4(2.1): 52 (1905) ♀♂, Type as above.
Cynoglossum geometricum Baker & C.H. Wright, in Oliver et al., Fl. Trop. Afr. 4(2.1): 52 (1905). Type: Nyasaland [Malawi] ♀♂ Mount Chiradzulu, no date, *A. Whyte s.n.* (K-image! lectotype, here designated). Notes: The specimen was selected as a lectotype by B.L. Burtt on the sheet but was never designated formally.
Paracynoglossum geometricum (Baker & C.H. Wright) R.R. Mill. in Notes Royal. Bot. Gard., Edinb. 41 (3): 478 (1984). ♀♂, Type: Same as above.
Cynoglossum coeruleum subsp. geometricum (Baker & C.H. Wright) S. Edwards in Fl. Ethiopia & Eritrea 5: 93 (2006), nom. inval.

##### Type.

Cameroon ♀♂, Mount Cameroon, Dec 1862, *Mann 2005* (K-image, lectotype! here designated; K-image [2 sheets] isolectotype!).

**Figure 9.**
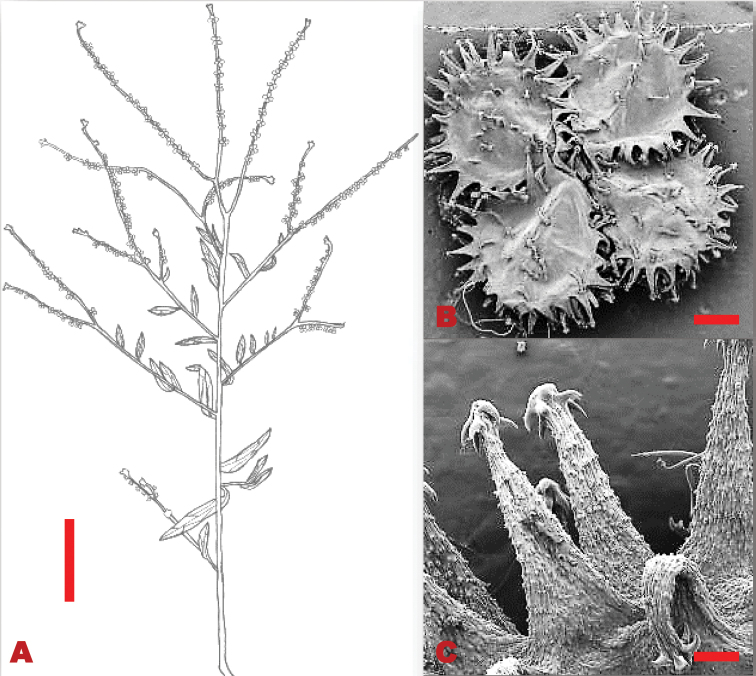
Vegetative and reproductive morphological features of Cynoglossumcoeruleumvar.mannii**A** line drawing of dichotomous branching of the fruit stalk **B** marginal and median line glochidia on the adaxial surface of a nutlet **C** Glochidia evenly sized. Voucher specimen: *T.B. Sikhakhane 440* (NH). Drawing scale bar: 7.5 mm. SEM scale bar: 2 mm (**B**); 200 µm (**C**).

Perennial, biennial, or annual herbs, 1.2 m in height. Basal leaves 90–190×28–56 mm, lanceolate-obtuse, softly hairy, deciduous, margins entire. Stem leaves 35–90×7–25 mm, lanceolate, apex acute, base acute to obtuse, covered with moderately stiff hairs margins entire. Trichomes bulbous based on the upper surface of the leaf, sometimes simple on the lower surface. Inflorescences terminal axillary cymes, few branches spreading dichotomously; pedicel 4–8 mm long, lengthens considerably in fruit. Calyx ca. 21 mm long, lobes ovate-oblong, adpressed-hairy outside, smooth inside, apex acute. Corolla white with pale blue throat; lobes ca. 2.1 mm in diameter, campanulate. Nutlets ovoid, 3–4×2.5–3.5 mm; glochidia more marginal and on the median line (Figure 9).

##### Phenology.

December to April.

##### Conservation status.

Least Concern (Raimondo et al. 2009).

##### Diagnostic characters.

This variety can be easily confused with *C.lanceolatum* due to the dichotomous branching of the inflorescence but can be distinguished from it by the distribution and density of the glochidia in the nutlets. The glochidia on the nutlets of Cynoglossumcoeruleumvar.mannii are more marginal and on the median line, whereas they are equally distributed around the whole nutlet in *C.lanceolatum*.

##### Distribution and habitat.

**Figure 10.**
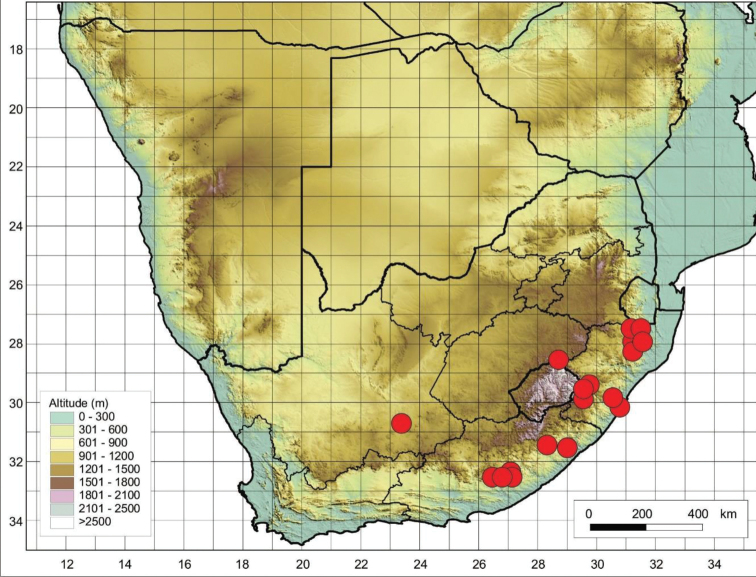
Known distribution of Cynoglossumcoeruleumvar.mannii in southern Africa based on the specimens examined.

This variety is endemic to South Africa where it is known only from KwaZulu-Natal and Eastern Cape Provinces (Figure 10). It is also reported from Malawi, Mozambique, Zambia, and Zimbabwe (Mill and Miller 1984). It is found in disturbed grassland and sandy areas.

##### Additional specimens examined.

South Africa. Kwazulu-Natal: 2731 (Louwsburg): Itala Nature Reserve (-AD), 10 Dec 1987, *M. Jordaan 7064* (NH); 9 Dec 1987, *A.G. Hutchings 2530* (NU); Zululand District Municipality Area, Abaqulusi Municipality Area, Tygerskloof Farm (-CD), 24 Jan 2012, *A.M. Ngwenya* and *D.G.A. Styles 4034* (NH); 7 Mar 2019, *A.N. Moteetee* and *L.K. Madika AL010* (JRAU). 2828 (Bethlehem): Royal Natal National Park (-DB), 17 February 1984, *O.M. Hilliard* and *B.L. Burtt 17658* (NBG; NU). 2831 (Nkandla): Nhlazatshe farm, (-AA), 4 Mar 1994, *T.B. Sikhakhane 440* (NH). 2929 (Underberg): Mpendhle District, Loteni Nature Reserve (-AD), 1 Feb 2001, *A.M. Ngwenya 1940* (NH), Loteni, upper reaches of river (-BC), 31 Mar 1984, *O.M. Hilliard 8218* (NU, PRE), Sipongweni Mountain (-CD), 20 Mar 1987, *O.M. Hilliard* and *B.L. Burtt 8249* (PRE). 2930 (Pietermaritzburg): On the Phezulu Game Estate, Botha’s Hill (-DC), 22 Jan 2005, *D. Styles 2280* (NH). 3030 (Port Shepstone): M. Stainbank’s farm, mid Illovo (-BB), 23 Dec 2008, *A. Young 942* (NU). Eastern Cape: 3023 (Britstown): Kamberg (-CC), 21 Mar 1983, *O.M. Hilliard 8208* (NU). 3128 (Umtata): Baziya Mission (-CB), 12 Feb 1981, *O.M. Hilliard* and *B.L. Burtt 13952* (NU); Nenga River, (-DB), 26 Oct 2001, *E. Cloete 6342* (GRA). 3226 (Fort Beaufort): Hogsback (-DB), Apr 1956, *R. Collett 9775* (GRA); Apr 1955, *A.R.H. Martin 9678* (GRA); Apr 1962, *A. Jacot Guillarmod 5544* (GRA); 12 Apr 1955, *L.M. Johnson 1152* (GRA); 4 Mar 1973, *M. Bradley 55* (GRA).

##### Taxonomic notes.

(i) *Cynoglossumgeometricum* was recorded in the southern African region (FSA) for the first time by Hilliard and Burtt (1986). Although the name seems to be accepted in the Flora of southern African region (FSA), for example, Germishuizen and Meyer (2003) and Burrows and Willis (2005), it is not the correct name for this taxon. The correct name is Cynoglossumcoeruleumsubsp.johnstoniivar.mannii, as synonimized by Verdcourt (1991). It is worth noting that the latter name is erroneously listed as C.coeruleumvar.mannii in websites such as Plants of the World Online (http://powo.science.kew.org/taxon/966730-1) and the World Flora Online (http://www.worldfloraonline.org/). Verdcourt (1991) relegated *C.johnstonii* to subspecies level under *C.coeruleum* and then transferred *C.mannii* to *C.coeruleum* as a variety of Cynoglossumcoeruleumsubsp.johnstonii. (ii) There are three sheets of *Mann 2005* in Royal Botanic Gardens, Kew (K), the one with the barcode number K000418935 is chosen as a lectotype because it displays most of the important characters of the species which can be used to distinguish this species from the rest, such as the inflorescence character and the branching pattern.

#### ﻿5. Cynoglossum hispidum

Taxon classificationPlantaeBoraginalesBoraginaceae

Thunb., Prodr. Plant. Cap. 1:34 (1794); Roemer and Schultes in Syst. Veg., ed. 15 bis 4: 79,761 (1819); C.H. Wright in F.C. 4(2):14(1904); Brand in Engl. Planzenr. 78 [4,252]:146 (1921).

Cynoglossum glomeratum Pursh in Fl. Amer. Sept. 2:729 (1813).
Type. United States of America ♀♂, Louisiana, no date, Bradbury s.n. (PH-image! holotype). Cynoglossumenerve Turcz. Bull. Soc. Imp. Naturalistes Moscou 1:259 (1840), E.Mey. ex DC., Prodr.10:154 (1846).
Type. South Africa. ♀♂, Eastern Cape, between Omcamwubo and Omcamcaba, no date, *Drѐge d* (HAL-image! lectotype, here designated; GDC-image! [3 sheets], isolectotype). [Note: The HAL specimen is chosen as a lectotype because the specimen displays the diagnostic characters of the species as described in the protologue].
Echinospermum enerve E.Mey. ex DC. Prodr. 10:154 (1846), nom. nud.

##### Type.

South Africa ♀♂, Precise locality unknown, Lange Kloof, *Thunberg 168* sub THUNB-UPS 3996 (UPS, microfiche! holotype).

**Figure 11.**
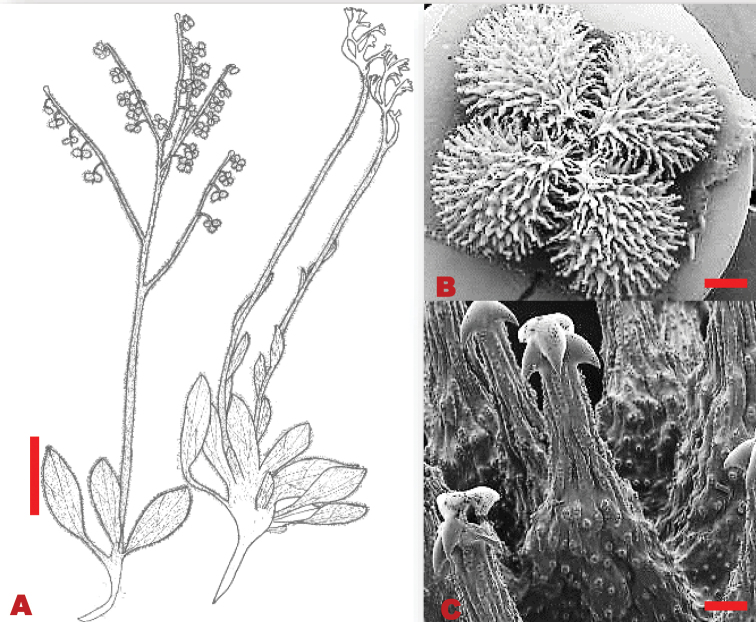
Vegetative and reproductive morphological features of *Cynoglossumhispidum***A** line drawing of obtuse shaped, rosette base leaves, terminal branched fruits, terminal flowers **B** densely packed with glochidia nutlets **C** Glochidia wide at the base, with multiangular tip. Voucher specimen: *S.P. Bester 12958* (PRE). Drawing scale bar: 7.5 mm. SEM scale bar: 5 mm; 100 µm (**C**).

Perennial or biennial herbs, 0.5–0.76 m in height. Basal leaves 80–250×15–25 mm, lanceolate-obtuse, densely pubescent, deciduous, margins entire. Stem leaves 60–80×5–12 mm, oblong-lanceolate, apex acute, base cuneate, covered with brittle hairs, margins entire. Trichomes bulbous based on the upper surface of the leaf, sometimes simple on the lower surface. Inflorescence terminal axillary cyme, branches spreading dichotomously; pedicel up to 20 mm long, lengthens considerably in fruit. Calyx ca. 5–10 mm long, lobes obtuse, outer surface packed with bulbous-based trichomes, apex acute. Corolla magenta; lobes ca. 5 mm in diameter, cruciform. Nutlets convex, 5–6×3–5 mm, highly pubescent; glochidia short and thick at the base, tips multiangular (Figure 11).

##### Phenology.

October to March.

##### Conservation status.

Least Concern (Raimondo et al. 2009).

##### Diagnostic characters.

The species can be confused with *C.lanceolatum* with which it shares a similar branching pattern of the inflorescences and upright brittle hairs covering the whole plant. However, the two differ in the colour of the corolla (magenta-purplish vs. white with blue throat in *C.lanceolatum*) and pedicel length (2 cm long opposed to less than 2 cm long in *C.lanceolatum*).

##### Distribution and habitat.

**Figure 12.**
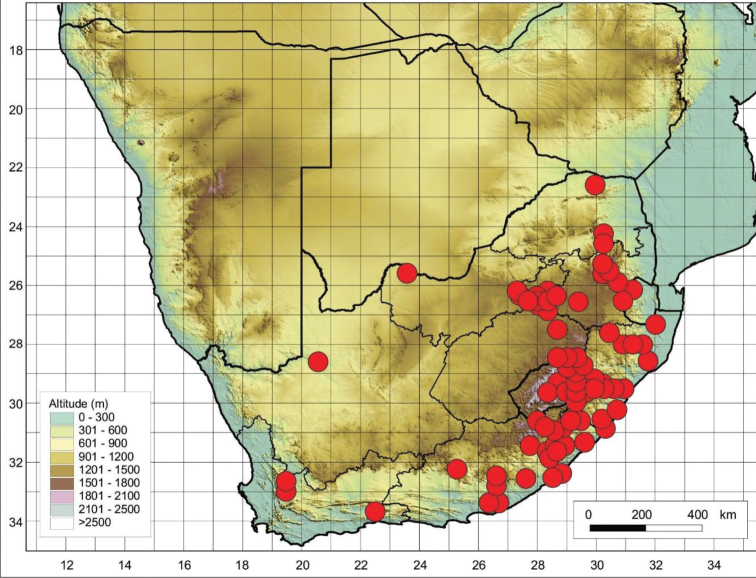
Known distribution of *Cynoglossumhispidum* in southern Africa based on the specimens examined.

This species is widely distributed across all provinces of South Africa. It can also be found in eSwatini and Lesotho (Figure 12). It mostly occurs in open grasslands, grassy slopes, woodland marshes, and disturbed areas like abandoned lands.

##### Additional specimens examined.

South Africa. Limpopo: 2229 (Waterpoort): Evelyn valley (-BD), Feb 1944, *RUC Biology Expedition 417* (GRA). 2430 (Tzaneen): Lekgalameetse Nature Reserve (-AA), 4 Dec 1985, *M. Stalmans 790* (PRE); Sekhukhune District, Leolo Mountains (-CA), 14 Mar 2007, *B. Sachse 471* (PRE). North-West: 2523 (Pomfret): Forbes (-DA), 29 Oct 1959, *B. Dlamini s.n.* (NH). 2627 (Potchefstroom): Goedgedacht (-AA), 1 May 1932, *J.D. Sutton 676* (PRE); Losberg, Elandsfontein (-BC), 11 Dec 1934, *J.J. Theron 725* (NH); Potchefstroom (-CA), 23 Nov 1946, *W.J. Louw 1525* (PRE). Gauteng: 2528 (Pretoria): Brooklyn (-CA), 16 Sep 1928, *A.O.D. Mogg 15247* (PRE); 6 Apr 2019, *A.N. Moteetee* and *L.K. Madika AL014* (JRAU); 1931, *A.O.D. Mogg 16601* (PRE); 30 Sep 1943, *A.O.D. Mogg 17001* (PRE); Doornpoort/ Hartbeesfontein (-CB), 24 Jan 2004, *S.P. Bester 4656* (PRE); Irene District (-CC), Oct 1929, *A.A. Obermeyer 27651* (PRE). 2628 (Johannesburg): Vereeniging District (-AC), 23 Nov 2007, *S.P. Bester 8268* (PRE); Suikkerbosrand Nature Reserve (-AD), 7 Apr 1970, *A. Lambrechts 265* (PRE). Mpumalanga: 2530 (Mashishing): Boschhoek (-AA), 16 Nov 1933, *R.S.N. Young A375* (PRE); Verlorenkloof Reserve, Welgedacht (-AD), 28 Nov 2008, *S.P. Bester 8679A* (PRE); Machadodorp, Farm Grootvlei (-CB), 21 Oct 1988, *P. Burgoyne 457* (PRE); Songimvelo Game Reserve (-DD), 10 Dec 1992, *M. Jordaan 2490* (PRE). 2629 (Bethal): Bethal (-AD), 14 Dec 2010, *R. Leendertz 9386* (PRE). 2630 (Carolina): Ermelo District, Spitskop (-CA), Dec 1915, *R. Potts 5007* (PRE). Northern Cape: 2820 (Kakamas): Krantzkop (-DA), Nov 1911, *J. Thode 4768* (NBG). Free State. 2728 (Frankfort): Farm Reitfontein (-BC), 28 Jan 1983, *E. Retief 1071* (PRE). 2828 (Bethlehem): Qwaqwa National Park (-BC), 22 Nov 1994, *P.C. Zietsman 2558* (NH); Golden Gate National Park (-DA), 12 Dec 1988, *Gertenbach* and *Groenewald 8929* (PRE); Witsieshoek (-DB), 10 Mar 2015, *S. Parbhoo 81* (NH). 2829 (Harrismth): Harrismith (-AC), 17 Mar 1981, *M.L. Jacobsz 3092* (PRE); 18 Nov 1978, *M.L. Jacobsz 1299* (PRE); Rensburgskop (-AD), 10 Dec 1962, *M.L. Jacobsz 199* (NBG; PRE). Kwazulu-Natal: 2730 (Vryheid): Oshoek District, Wakkerstroom (-AD), 19 Nov 1962, *N.J. Devenish 954* (PRE); Klipspruit Dam (-DD), 10 Feb 2005, *S.P. Bester 6540* (NH, PRE). 2731 (Louwsburg): Zululand District municipality (-CD), 29 Oct 2000, *T. Edwards* and *C. Potgieter s.n.* (NU); 26 Jan 2012, *A.M. Ngwenya* and *D.G.A. Styles 4085* (NH). 2732 (Ubombo): Ingwavuma (-AA), 22 Nov 1969, *E.J. Moll 4680* (NH, PRE). 2829 (Harrismith): Cathedral Peak (-CC), 10 Nov 1956, *D.J.B. Killick 1115* (PRE); 13 Oct 1984, *J. Scott 251* (NH). 2830 (Dundee): Dundee District (-AA), 26 Nov 1964, *N.E. Shirley s.n.* (NU); Klipriver District, Elandslaagte (-CD), 23 Oct 1964, *N.E. Shirley s.n.* (NU). 2831 (Nkandla): Empangeni (-DB), 9 Jul 1965, *H.J.T. Venter 1917* (PRE). 2929 (Underberg): Giants Castle Game Reserve (-AB), 8 Feb 1966, *W.R. Trauseld 575* (PRE); (-AD), 8 Nov 2001, *T.R. Green 1217* (NU); Mpendhle District (-BC), 5 Jan 1983, *O.M. Hilliard* and *B.L. Burtt 16218* (PRE); Underberg District (-CB), 3 Feb 1975, *O.M. Hilliard* and *B.L. Burtt 7942* (NU); 3 Feb 1976, *O.M. Hilliard* and *B.L. Burtt 8905* (NU); 24 Mar 1977, *O.M. Hilliard* and *B.L. Burtt 9818* (NU); 11 Jan 1978, *M.A. Rennie 911* (NU); 27 Jan 1982, *M.A. Rennie 1307* (NU); 13 Feb 1983, *O.M. Hilliard* and *B.L. Burtt 17234* (NU); 6 Jan 1984, *O.M. Hilliard* and *B.L. Burtt 17290* (PRE); Underberg District (-CC), 19 Jan 1984, *O.M. Hilliard* and *B.L. Burtt 17346* (NU); 9 Jan 1986, *O.M. Hilliard* and *B.L. Burtt 18998* (NU); Hlogoma Mountain (-DC), 30 Nov 2015, *S.M. Berruti 513* (NH); Donnybrook (-DD), 7 Nov 2013, *D.G.A Styles 4579* (NH); 8 Mar 2019, *A.N. Moteetee* and *L.K. Madika AL011* (JRAU). 2930 (Pietermaritzburg): Lidgetton (-AC), 23 Mar 1920, *A.O.D. Mogg 6894* (PRE); 29 Sep 1964, *E.J. Moll 1036* (NU, PRE); 13 Jan 1988, *B. Grove 98* (NU); Umgeni River (-CA), 20 Oct 1984, *J. Manning 538* (NU); 8 Mar 2019, *A.N. Moteetee* and *L.K. Madika AL013* (JRAU); Ukulinga farm (-CB), 8 Mar 1982, *J.C. Manning 212* (NU); Nagle Dam (-DA), 15 Sep 1957, *M.J. Wells 1676* (NU); Cliffdale road (-DC), 17 Aug 2002, *D.G.A. Styles 9141* (NU); 30 Jul 2003, *P. Wragg 205* (NU),7 Nov 2014, *D.G.A. Styles 4925* (NH). 2931 (Stanger): Groutville (-AD), 14 Oct 1965, *E.J. Moll 2500* (NU, PRE). 3029 (Kokstad): Insizwa (-CA), 24 Feb 1972, *R.G. Strey 10827* (NU); Kokstad District (-DA), 1968, *C.J. Piek 53* (NH); 25 Feb 1978, *T.A. Coleman 985* (NH); no date, *F.M. Getliffe* and *T. Edwards 1266* (NU). 3030 (Port Shepstone): M. Stainbank’s farm, mid Illovo (-BB), 24 Sep 2009, *A. Young 1155* (NU); Umzinto District (-BC), 3 Sep 1983, *K. Balkwill* and *J.C. Manning 828* (NU); 20 Oct 1997, *A.M. Ngwenya 1538* (NH); Port Shepstone (-CB), 3 Oct 1937, *A.O.D. Mogg 13873* (PRE); (-CD), 20 Sep 1955, *S. McNeil 142* (NU). 3030 (Umzinto): Vernon Crookes Nature Reserve (-BC), 3 Sep 1983, *K. Balkwill* and *J.C. Manning 808* (NU); 29 Sep 1984, *C.F. Kennedy 32* (NU). Western Cape: 3219 (Cape Town): Rivierground in pypsteelbosse (-CC), 18 Dec 1980, *W.J. Hanekom 2600* (PRE); Kouebokkeveld Berge, (-CC), 24 Nov 1998, *W.J. Hanekom 3120* (NBG). 3322 (Oudtshoorn): Outeniqua Mountains (-CC), 9 Nov 1986, *J.H.J Vlok 1693* (PRE). Eastern Cape: 3027 (Barkly East): Farm Faskally near England (-DA), 9 Nov 1995, *J.E. Victor 1586* (PRE); Ben McDui (-DC), 6 Jan 1997, *T. Dold* and *M. Cocks 3495* (GRA). 3028 (Umtata): Maclear District (-CA), 16 Jan 2016, *S.P. Bester 13207* (PRE); Maclear (-CC), 7 Nov 1993, *S.P. Bester 1535* (NH); Kloof (-DD), 28 Feb 1946, *R. Story 950* (PRE). 3128 (Umtata): Hill above Mhlanfane Forest Station (-BC), 31 Jan 1983, *O.M. Hilliard* and *B.L. Burtt 16341* (NU); Lady Frere, Engcobo (-CC), 25 Nov 1990, *E. Cloete 590* (NH); (-DB), 21 Oct 1953, *G.C. Theron 1604* (PRE); Elliotdale District (-DC), 11 Jul 1966, *J.L. Gordon-Gray 529* (NH). 3129 (Umtata): Baziya, Tembuland (-CC), no date, *Baur 257* (K-image); Blesbok flats Cathcart Div. Cape, 1838, *Drége s.n*. (K-image); between St John’s River and Umtsikaba River, Pondoland, 1838, *Drége s.n* (K-image!). 3225 (Elandsfontein): Elandsfontein (-AA), 13 Dec 2005, *S.P. Bester 6347* (GRA, PRE). 3226 (Fort Beaufort): Along gravel road to Sada off the R67 (-BD), 8 Feb 1995, *J.E. Victor* and *D.B. Haare 392* (PRE); Mpofu Game Reserve (-DA), 28 Feb 2006, *C.L. Bredenkamp 3338* (PRE); Menzieberg, Amatole Mountains (-DB), 6 Jan 1986, *P.B. Phillipson 1185* (GRA), Fort Beaufort Road, 2 miles (3.22 km) from Alice (-DD), 22 Oct 1939, *M.H. Giffen 264* (PRE). 3227 (Stutterheim): Stutterheim (-AD), 5 Dec 1942, *J.P.H. Acocks 9399* (PRE); Pirie Mission (-CC), exact date not provided 1888, *T.R. Sim 20419* (NU, PRE); 5 Aug 2014, *S. Mgcuwa 117* (GRA); King William’s Town (-DB), Nov 1891, *H.G. Flanagan 1193* (PRE). 3326 (Grahamstown): Faraway (-AD), 4 Dec 1980, *A. Jacot Guillarmod 8473* (GRA); Nov 1942, *E. Archibald 669* (GRA); Alexandria (-CB), 10 Aug 1953, *S. Johnson 691* (GRA); 11 Feb 1953, *W. Marais 185* (PRE). 3327 (Peddie): Peddie District (-AC), 5 Nov 1993, *T. Dold* and *A. Booi 481* (GRA).

eSwatini. 2631 (Mbabane): Forbes reef (-AA), 29 Oct 1959, *B. Dlamini s.n*. (NBG).

Lesotho. 2928 (Marakabei): Ntiboho valley (-AC), 2 Jan 1947, *A. Jacot Guillarmod 279* (GRA). 2929 (Underberg): Thaba Ntšo, Sehlabathebe National Park (-CC), 4–14 Jan 1973, *Jacot Guillarmod*, *Getliffe* and *Mzamane 65* (PRE); 13 Feb 1976, *A. Beverly 471* (PRE).

Unknown localities: Feb 1895, *Maurice* and *Evans 474* (NH); Feb 1939, *J. Wylie 30120* (NH); Apr 1943, *B. Fischer 464* (NU); 15 Dec 1943, *W.F. Barker 2796* (NBG); 18 Nov1952, *H.B. Gilliland 26862* (PRE); 18 Dec 1966, *R.G. Strey 7047* (NH); Mar 2000, *T. Edwards* 2088 (NU).

##### Taxonomic notes.

Brand (1921) included *C.hispidum* as a synonym of *C.lanceolatum*. However, the two species have notable variations especially in the colour of the corolla (magenta-purplish vs. white with blue throat in *C.lanceolatum*) and pedicel length (2 cm long opposed to less than 2 cm long in *C.lanceolatum*).

#### ﻿6 Cynoglossum lanceolatum

Taxon classificationPlantaeBoraginalesBoraginaceae

Forssk in Fl. Aegypt. Arab: 41 (1775).

Cynoglossum hirsutum Thunb., Prodr. Pl. Cap.: 34 (1794).
Type: South Africa ♀♂, Precise locality unknown: Roggerveld, *C.P. Thunberg 168*, sub THUNB-UPS 3995 (UPS-microfiche! holotype).
Cynoglossum micranthum Desf.: 220 (1804), nom. nud.
Cynoglossum canescens Willdenow in Enum. Pl.: 180 (1809).
Type: ♀♂, Precise locality unknown, *C.L. Willdenow*, *s.n*. (B-W,-image, lectotype, designated by Kӧnig et al. 2015).
Cynoglossum racemosum Roxb. in Fl. Ind.: 2:6 (1824), nom. illeg.
Echinospermum paniculatum E.Mey. ex. A.DC., Prodr. [A.P.de Candolle] 10: 149,143 (1846). ♀♂, Type: same as above.
Paracynoglossum lanceolatum (Forssk.) R.R.Mill. in Notes Roy. Bot. Gard. Edinb.: 474 (1984). ♀♂Type: same as above.

##### Type.

Yemen, Al Hadiyah, Mar 1763, *P. Forsskal 312* (C-image! holotype).

**Figure 13.**
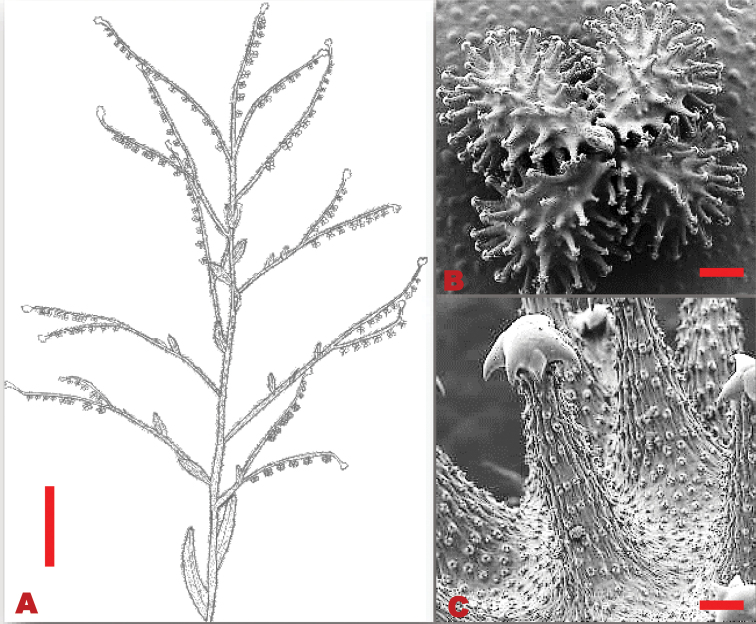
Morphological characters of *Cynoglossumlanceolatum***A** line drawing of the fruiting branch **B** Glochidia evenly distributed on the adaxial surface of the nutlet **C** Glochidia uniform size, with multiangular tip. Voucher specimen: *S.P. Bester 4653* (PRE). Drawing scale bar: 7.0 mm. SEM scale bar: 1 mm (**B**); 100 µm (**C**).

Annual or biennial herbs, 0.5–0.9 m in height, covered with simple hairs. Basal leaves 85–180×15–23 mm, lanceolate-obtuse, blade elliptic, softly hairy, deciduous. Stem leaves 40–65×8–18 mm, lanceolate, apex acute, base cuneate, covered with moderately stiff hairs. Trichomes brittle, simple on both leaf surfaces. Inflorescence dichotomously branched axillary cyme, pedicel 1–1.5 mm long. Calyx ca. 1–1.5 mm long, lobe ovate-obtuse, pubescent on the outer surface, inner surface glabrous, apex acute. Corolla white with pale blue throat; lobes ca. 1×1 mm, campanulate. Nutlets ovoid-convex, 3–4×2.5–3.5 mm, fully pubescent; glochidia equally thick and long (Figure 13).

##### Phenology.

August to May.

##### Conservation status.

Least Concern (Raimondo et al. 2009).

##### Diagnostic characters.

Amongst the southern African species, *C.lanceolatum* is similar and possibly related to Cynoglossumcoeruleumsubsp. johnstoniivar.mannii with which it shares the branching pattern of the inflorescence, flower colour, and nutlet size. The two species can be distinguished by the density of the glochidia on the nutlets and distribution. *Cynoglossumlanceolatum* nutlets are completely covered with glochidia, whereas in Cynoglossumcoeruleumsubsp. johnstoniivar.mannii glochidia tend to be more marginal and acentric.

##### Distribution and habitat.

**Figure 14.**
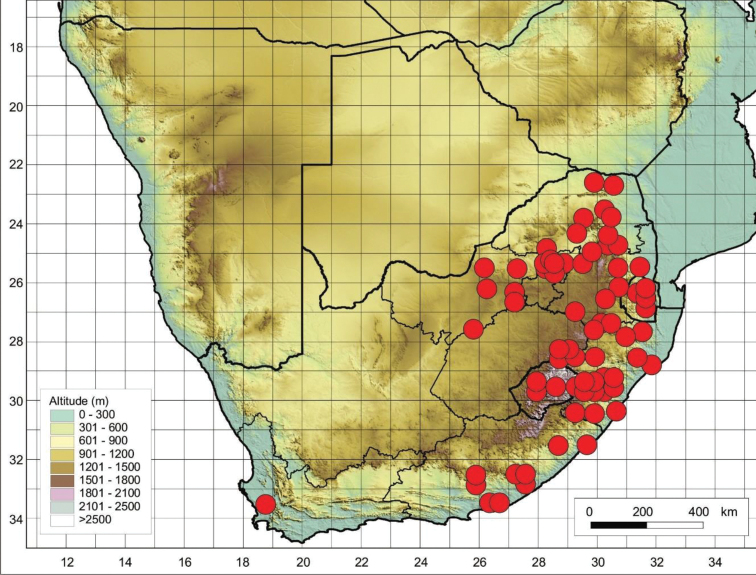
Known distribution of *Cynoglossumlanceolatum* in southern Africa based on the specimens examined.

*Cynoglossumlanceolatum* originates from Yemen (Kӧnig et al. 2015) but reported from Africa (Ge-Ling et al. 1995), Pakistan, India (Joshi 2016), the Mediterranean, and throughout Asia (Verdcourt 1991) and Madagascar (Kӧnig et al.2015). In South Africa it occurs widely in all provinces, it also occurs in eSwatini and Lesotho (Figure 14). It is a widespread species that grows in disturbed habitats throughout parts of Africa.

##### Additional specimens examined.

South Africa. Limpopo: 2229 (Waterpoort): Wylies Poort (-DD), 17 Oct 1938, *J.P.M. Acocks 1295* (PRE). 2230 (Messina): Sibasa District (-CD), 21 Jun 1977, *G. Hemm 163* (PRE). 2329 (Polokwane): Polokwane Nature Reserve (-CD), 12 Jan 1979, *Bredenkamp* and *Van Vuuren 307* (PRE). 2330 (Tzaneen): Letaba (-CA), 2 Apr 1958, *J.C. Scheepers 219* (PRE); Woodbush Forest Reserve (-CC), Jan 1923, *H. Wafen 22973* (PRE); 1 Feb 2019, *A.N. Moteetee* and *L.K. Madika AL07* (JRAU). 2428 (Modimolle): Waterberg, Kwaggasnek-Alma Road (-CC), 13 Feb 1981, *K.L. Immelman 118a* (PRE). 2429 (Zebediela): Zebediela (-AA), 5 Feb 1967, *B.J. Huntley 1033* (PRE); 2430 (Pilgrim’s Rest): Sekhukhune District Municipality, Leolo Mountain, SE of Tama Kgoshi (-CA), 14 Mar 2007, *B. Sachse 466* (PRE). North-West: 2526 (Zeerust): Zeerust along road to Koster (-CA), 4 Feb 1976, *F. van der Meulen 597* (PRE). 2527 (Rustenburg): Rustenburg (-CA), 27 Dec 1949, *S.M. Johnson 854* (NBG). 2626 (Lichtenburg): Municipal Caravan Park (-AA), 19 Mar 1978, *A. Balsinhas* and *Harding 3303* (PRE). 2627 (Potchefstroom): Carletonville, A. Bailey Nature Reserve (-AD), Apr 1983, *S. van Wyk 180* (PRE); Mooiriver (-CA), 9 Mar 1984, *B. Ubbink 1257* (PRE). 2724 (Taung): West of Harz River near Taung, 120 km north of Kimberley (-DB), 11 Feb 1948, *R.J. Robin 3628* (PRE). Gauteng: 2528 (Pretoria): Brooklyn (-CA), 20 May 1915, *C.A. Smith 191* (PRE); 24 May 1915, *C.A. Smith 194A* (PRE); 10 Dec 1915, *A.O.D. Mogg 11846* (PRE); Nov 1925, *C.A. Smith 1220* (PRE); Doornpoort, Airport Road (-CA), 26 Oct 2009, *S.P*. *Bester 9734* (PRE); 14 Nov 2018, *A.N. Moteetee* and *L.K. Madika AL01* (JRAU); Doornpoort/ Hartbeesfontein (-CB), 24 Jan 2004, *S.P. Bester 4653* (PRE); Brooklyn (-CC), 11 Apr 1920, *K.A. Landsdell 864* (PRE). 2628 (Johannesburg): Randburg, Lanseria (-AA), 20 Nov 2018, *A.N. Moteetee* and *L.K. Madika AL02* (JRAU); Suikkersbosrand (-CB), 27 Dec 1971, *G.J. Bredenkamp 594* (PRE). Mpumalanga: 2429 (Zebediela): Mashishing District (-DD), 8 January 1939, *Barnard* and *Mogg 860* (PRE). 2430 (Pilgrim’s Rest): Malta near Marinella (-AA), 16 Aug 1984, *M. Stalmans 114* (PRE); Ohrigstad Dam Nature Reserve (-DC), 22 May 1973, *N. Jacobsen 2861* (PRE). 2529 (Emalahleni): Loskop Dam (-AD), 12 Jan 1967, *G.K. Theron 1129* (PRE). 2530 (Mashishing): Witklip (-BD), 4 Dec 1973, *J.P. Kluge 359* (PRE); Lowveld Botanical Gardens (-BD), 18 Dec 1974, *E.J. Van Jaarsveld 199* (NBG); Waterval Onder, Ntsinini (-CD), 26 Dec 2008, *K.W. Grieve 250* (PRE); 23 Jan 2011, *K.W. Grieve 342* (PRE); Barberton (-DD), 24 Jan 2000, *J.J. Meyer 2603* (PRE). 2531 (Beersrust): White River District (-AC), Apr 1983, *W. Jacobsen 5363* (PRE). 2630 (Carolina): Chrissiesmeer (-AC), 5 Jan 1972, *G.K. Theron 2402* (PRE); Chrissiesmeer, on road to Lochiel (-BA), 6 Mar 1986, *M. Crosby 273* (PRE). 2730 (Vryheid): Farm Doornhoek along Assegaai river (-AA), 15 Dec 2006, *S.J. Siebert 3246* (PRE). Free State: 2729 (Volksrust): Vrede (-CB), 5 Feb 1987, *L. Smook 6455* (PRE). 2828 (Bethlehem): Golden Gate National Park (-DA), Jan 1963, *L.C.C. Liebenberg 6831* (PRE); Witsieshoek (-DB), Mar 1917, *H.A. Janod 17317* (PRE). 2829 (Harrismith): Harrismith District (-AC), 8 Mar 1974, *M.L. Jacobsz 2036* (PRE). Kwazulu-Natal: 2729 (Utrecht): Volksrust (-BD), Jan 1928, *C.A. Smith 5730* (PRE). 2730 (Vryheid): Oshoek District, Wakkerstroom (-AB), 26 Dec 2002, *S.J. Siebert* and *F. Siebert 2279* (PRE); Vryheid Nature Reserve (-DC), 23 Feb 1988, *C.J. Youthed 11* (NH); 7 March 2019, *A.N. Moteetee* and *L.K. Madika AL09* (JRAU). 2731 (Louwsburg): Louwsburg District, Itala Nature Reserve (-CB), 15 Jan 1978, *D.J. Mcdonald 443* (PRE). 2828 (Bethlehem): Crocodile River (-DB), 16 Dec 1893, *R. Schlechter 3980* (NH); Royal Natal National Park (-DB), 17 Feb 1984, *O.M. Hilliard* and *B.L. Burtt 17662* (NU); Tugela gorge (-DB), 6 Feb 1982, *O.M. Hilliard* and *B.L. Burtt 15454* (PRE); Tugela valley (-DD), 14 Feb 1926, *A.J.W. Bayer 47* (PRE). 2829 (Harrismith): Ladysmith (-DB), 1 Nov 1965, *N.E. Shirley s.n.* (NU). 2831 (Nkandla): Nkandla, Ferncliff Nature Reserve (-CA), 14 Feb 1994, *H. Kennedy 533* (NU); University of KwaZulu-Natal (-DD), 27 May 2003, *S.J. Siebert 2333* (NH). 2929 (Underberg): Loteni Nature Reserve (-AD), 13 Dec 1978, *M.L. Jacobsz 3937* (PRE); Mpendhle District (-BC), 24 Dec 1978, *O.M. Hilliard* and *B.L. Burtt 11804* (NU, PRE); Polela District (-CB), 6 Apr 1974, *M.A. Rennie 545* (NU); Waterfall (-DD), Mar 2002, *T. Edwards 2731* (NU). 2930 (Pietermaritzburg): Victoria (-AA), 12 Apr 1955, *S.M. Johnson 1152* (NH); Tweedie (-AC), 31 Dec 1927, *Forbes 293* (NH); 28 Feb 1982, *K.L. Immelman 260* (PRE); Loskop District (-AD), 11 Feb 1946, *B.L. Howlett 80* (NH); Pietermaritzburg (-CB), 29 Feb 1976, *M. Grice s.n.* (NU); 14 Oct 1987, *Renecken 6* (GRA); Botanic Garden Estate (-CB), 8 Dec 1991, *D.G. Stielau 135* (NH, PRE); Cottingham (-CC), 23 Mar 1969, *R.G. Strey 8420* (NU); Durban Road (-CD), 19 Nov 1950, *J.G. Lawn 1820* (NH); Umgeni Water Board, (-DA), 20 Oct 1984, *J. Manning 536* (NU); Inanda (-DB), Dec, *J. Medley-Wood 370* (NH); 25 Nov 1983, *H.H. Hilger 35* (PRE); Pinetown District (-DD), 28 Nov 1913, *J.M. Wood 12373* (NU). 3029 (Kokstad): Harding, Victoria East (-CA), 30 Apr 1955, *G.L. Lewis 4914* (PRE); Harding, Alfred District (-DB), 2 Mar 1983, *O.M. Hilliard* and *B.L. Burtt 16755* (NU). 3030 (Port Shepstone): Dumisa (-AD), 9 Dec 1992, *A.M. Ngwenya 1071* (NH). Western Cape: 3318 (Cape Town): Zondagsfontein (-DC), Dec-Mar 1930–1, *J. Thode A2838* (NH); 18 Oct 1943, *B.S. Fischer 498* (NU). Eastern Cape: 3128 (Mjika): Mahlahlane Forest (-BC), 6 Mar 1985, *A. Hutchings 1590* (GRA). 3129 (Port St. John’s): Kloof above Port St. Johns (-DA), 31 Jan 1936, *M.C. Gillett 1257* (PRE). 3226 (Fort Beaufort): Amatole Mountain (-DB), 1 May 1986, *P.B. Phillipson 1492* (PRE); Fort Beaufort (-DD), 21 Mar 1977, *G.E. Gibbs Russell 3735* (GRA). 3326 (Grahamstown): Grahamstown Nature Reserve (-BC), Feb 1917, *J.C. Jane 17131* (PRE); 1 Apr 1952, *A.R.H. Martin 9466* (GRA); Featherstone Kloof (-BC), 18 Jul 2001, *C.J. Kayombo* and *A.A. Merti 3719* (GRA). 3227 (Stutterheim): Stutterheim District (-CB), 28 Jan 1979, *O.M. Hilliard* and *B.L. Burtt 12427* (NU); Kababu Hills (-CA), 5 Feb 1936, *M.C. Gillett 1325* (PRE); East London (-DB), 11 Dec 2001, *J.J. Meyer 4067* (PRE).

eSwatini. 2631 (Hhoho): Hhoho District, Masilela area on Maphalaleni road along Mucucene Hills (-AB), 27 Jan 1994, *G. Germishuizen 7173*, *7174* (PRE); 24 Jan 1994, *S.R. Hobson 2048* (PRE); Hlambanyathi valley (-AC), 27 Nov 1954, *R.J. Compton 24864* (NBG); Mbabane (-AC), 14 Jan 1955, *R.J. Compton 24834* (PRE).

Lesotho. 2927 (Maseru): Monethi’s, Berea (-BB), 1 Jan 1957, *A. Jacot Guillarmod 1910* (PRE); Mountain Road (-BD), Mar 1977, *M. Schmitz 7317* (PRE). 2928 (Marakabei): Loskop (-AB), 11 Apr 1980, *B.N. Ubbink 974* (NH); Molika-lika (-AC), 7 Jan 1954, *A. Jacot Guillarmod 1674* (PRE). 2929 (Underberg): Thaba Ntšo, Sehlabathebe National Park (-CC), 4–14 Jan 1973, *Jacot Guillarmod*, *Getliffe*, and *Mzamane 142* (PRE).

Unknown localities: No locality details, 30 Jan 1948, *B.S. Fischer 1426* (NU).

##### Taxonomic notes.

The specimen in the National Herbarium Pretoria (PRE) collected in the Doornpoort area in Gauteng Province (Voucher number: *S.P*. *Bester 9734* (PRE)) belongs to *C.lanceolatum* and not to *C.amabile* since it has characters which are typical of this species, i.e., white corolla with a blue throat instead of bluish-purple corolla and divaricately branched instead of clustered at the apex.

#### ﻿7. Cynoglossum obtusicalyx

Taxon classificationPlantaeBoraginalesBoraginaceae

Retief & A.E. van Wyk in S. Afr. J. Bot. 62(3): 169–172 (1996).

##### Type.

South Africa ♀♂, Western Cape, Worcester (3319): 20 km E. of Ceres (-AD), 21 Oct 1958, *Acocks 19893* (PRE-image! holotype; NBG, isotype).

**Figure 15.**
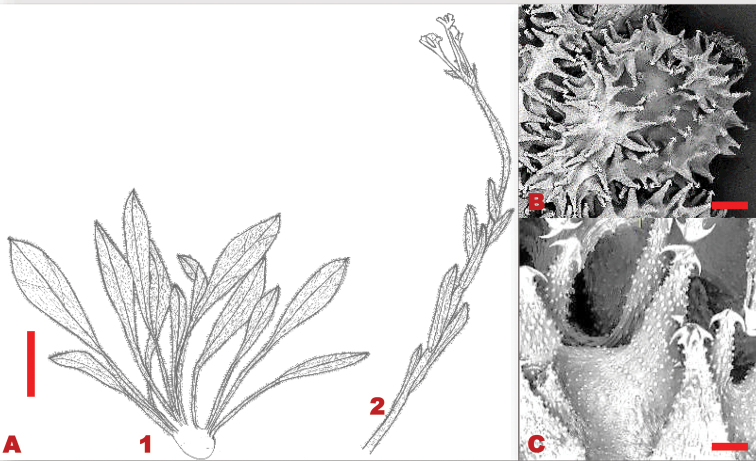
Vegetative and reproductive morphological features of *C.obtusicalyx***A** 1. base leaves 2. stem with alternate stem leaves and terminal flowers **B**SEM micrograph of a nutlet **C**SEM micrograph of glochidia around the nutlet. Voucher: *J.P.H. Acocks 8509* (PRE). Drawing scale bar: 7.0 mm. SEM images scale bar: 1 mm (**B**); 200 µm (**C**).

Perennial or biennial herbs, ca. 0.45 m in height. Basal leaves 175–250×5–18 mm, obtuse, densely pubescent, persistent, margins entire. Stem leaves 50–65×5–10 mm, obtuse-lanceolate, apex acute, base cuneate, soft hairs. *Trichomes* woolly, soft, non-bulbous based, unicellular, long, thin. Inflorescence dichotomously branched terminal cymes, pedicel 5–10 mm long, lengthens considerably in fruit. Calyx ca.1.0–1.6 mm long, lobe oblong, densely pubescent, apices broadly obtuse-truncate shaped. *Corolla* pale blue; lobes 5×7 mm diameter, ovate. Nutlets ovoid-convex, 2–4×3–4 mm, densely echinulate with glochidia (Figure 15).

##### Diagnostic characters.

The stems and leaves of *Cynoglossumobtusicalyx* are densely covered with soft, woolly trichomes which makes it similar to *Cynoglossumalticola*. However, the two species differ in flower colour and size (deep blue corolla, 4×3 mm vs. pale blue corolla, 5×7 mm in *C.obtusicalyx*). *Cynoglossumobtusicalyx* has pale blue flowers with corolla 5–7 mm long whereas *C.austroafricanum* has bright blue flowers with corolla 2.75–4.25 mm long. The two species also show variation in trichome texture and density, i.e., sparsely hairy with short, stiff hairs on both the stem and leaves in *C.austroafricanum* vs. densely arranged long, woolly hair on both the stem and leaves in *C.obtusicalyx*.

##### Phenology.

October to February.

##### Conservation status.

Least Concern (Raimondo et al. 2009).

##### Distribution and habitat.

**Figure 16.**
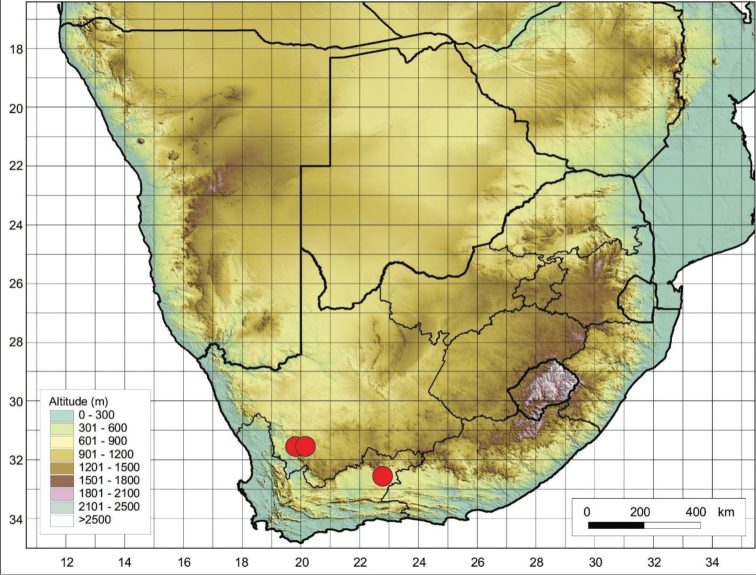
Known distribution map of *C.obtusicalyx* in southern Africa based on the specimens examined.

*Cynoglossumobtusicalyx* is endemic to South Africa where it has been recorded from Calvinia, Worcester, and Beaufort West in the Northern and Western Cape Provinces (Figure 16), it occurs in mountainous areas, often growing on screes below cliffs.

##### Additional specimens examined.

South Africa. Northern Cape: 3119 (Calvinia): Calvinia District (-BD), 22 Sep 1955, *J.P.H. Acocks 8509* (PRE); 22 Sep 1955, *D.A Leistner 394* (PRE; NBG). Western Cape: 3222 (Beaufort West): Mountain View farm (-BD), 17 Apr 1978, *G. Gibbs Russell* and *R. Hermann 4272* (PRE).

#### ﻿8. Cynoglossum spelaeum

Taxon classificationPlantaeBoraginalesBoraginaceae

Hilliard & B.L.Burtt in Notes Roy. Bot. Gard., Edinburgh 37(2): 287 (1979).

Cynoglossum basuticum Weim.ex Guillarmod, Fl. Lesotho 233 (1973), nom. nud.

##### Type.

South Africa ♀♂, KwaZulu-Natal, Underberg (2929): Underberg District, Cobham Forest Station, Polela valley (-CD), 20 Mar 1977, *O.M. Hilliard* and *B.L. Burtt 9728* (E, holotype image! K-image!, NU-image! PRE-image! [2 sheets] isotype).

**Figure 17.**
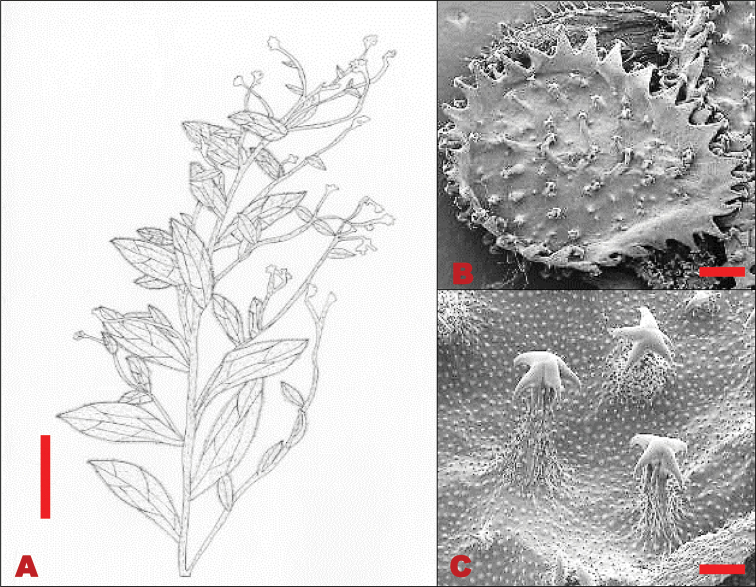
Morphological features of *C.spelaeum***A** line drawing of a fruiting branch showing stem with alternate, sessile, spathulate leaves **B**SEM micrograph of nutlet showing more marginal and acentric glochidia **C** close-up SEM micrograph showing glochidia with multiangular hooks. Voucher specimen: *A. Nicholas* and *B. Isaacs 1965* (PRE). Drawing scale 8.0 mm. SEM images scale bars: 1 mm (**B**); 200 µm (**C**).

Perennial herbs, ca. 0.4–0.5 m in height. Basal leaves 130–235×25–35 mm, spathulate-obtuse, soft hairs, deciduous, margins entire. Stem leaves 40–80×10–25 mm, obtuse shaped, dark green adaxial surface, grey green abaxial surface, smooth margins, soft hairs, sparsely packed. Trichomes unicellular, simple on both leaf surfaces, soft. Inflorescence corymbose panicle; threadlike pedicel 5 mm long. Calyx ca. 1.5–2.0 mm long, lobes lanceolate-oblong, softly hairy on the outer surface, inner surface smooth, apex acute. Corolla white; lobes ca. 3×3 mm diameter, obtuse. Nutlets ovoid, ca. 4×5 mm; glochidia more marginal and acentric, marginal glochidia are longer compared to the acentric glochidia (Figure 17).

**Figure 18.**
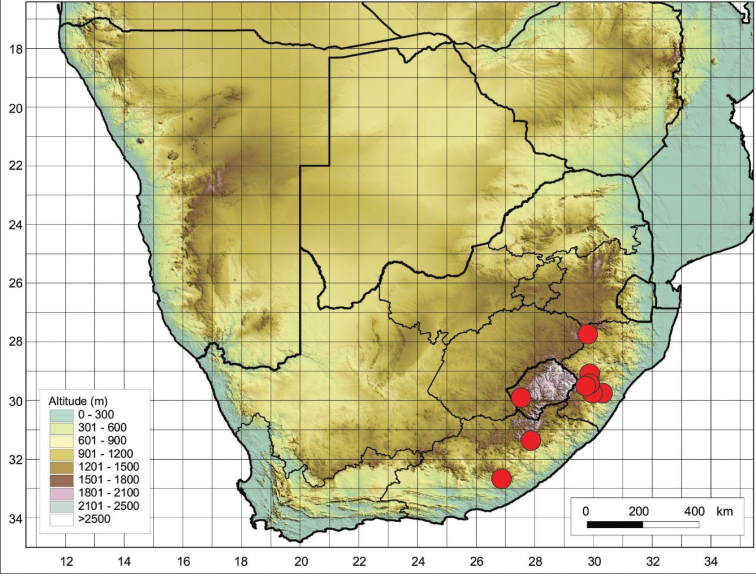
Known distribution of *Cynoglossumspelaeum* in southern Africa based on the specimens examined.

##### Phenology.

December to March.

##### Conservation status.

Least Concern (Raimondo et al. 2009).

##### Diagnostic characters.

This species is characterised by leaves that are sparsely covered with hairs that have a softer feel, which distinguishes it sharply from all the other southern African species which have either brittle or woolly hairs. The abaxial leaf surface has a grey-greenish appearance which is also a unique character of this species.

##### Distribution and habitat.

The species is distributed in South Africa (Eastern Cape, Free-State, and KwaZulu-Natal Provinces) and Lesotho (Figure 18). It grows in loose sandy soil at the edge of an overhang.

##### Additional specimens examined.

South Africa. Kwazulu-Natal: 2729 (Utrecht): Ncandu State Forest (-DC), 8 May 1984, *A. Nicholas* and *B. Isaacs 1965* (PRE). 2929 (Underberg): Allandale, Lion’s River District (-BC), 24 Jan 1978, *O.M. Hilliard* and *B.L. Burtt 11258* (NU); Giant’s Castle Nature Reserve (-BC), 07 Jan 1988, *A. Abbott 4064* (NH); 3 Feb 1976, *O.M. Hilliard* and *B.L. Burtt 8904* (NU); 2 Jan 1978, *O.M. Hilliard* and *B.L. Burtt 11172* (NU); 20 Nov 1985, *O.M. Hilliard* and *B.L. Burtt 18242* (NU, PRE); Bulwer District (-DD), 8 Jan 1974, *M.A. Rennie 507* (NU); 8 Mar 2019, *A.N. Moteetee* and *L.K. Madika AL012* (JRAU). Eastern Cape: 3127 (Lady Frere): Elliot District (-BB), 22 Jan 1979, *O.M. Hilliard* and *B.L. Burtt 123427* (NU). 3226 (Fort Beaufort): Amatole Mountain (-DB), 16 Feb 1986, *P.B. Phillipson 1294* (PRE).

Lesotho. 2927 (Maseru): Laikopile Mountain (-CD), Jan 1918, *A. Dieterlen 40799* (PRE).

##### Taxonomic notes.

*Cynoglossumspelaeum* is quite distinctive among the southern African species having uniquely shaped (i.e., spatulate to obtuse) and coloured (i.e., deep green adaxial surface, grey green abaxial surface) leaves, that almost seem leathery but contain few soft trichomes. It is also the only species among the southern African species that has completely white flowers. Although Hilger et al. (2015) indicated that the species does not belong to genus *Cynoglossum*, they did not elaborate the reasons for the exclusion. Nonetheless, the species fits the generic description of four glochidiate nutlets that are adapted for zoochory. In addition, preliminary molecular data (Madika 2020), showed a close relationship between C.coeruleumsubsp.johnstoniivar.mannii, *C.lanceolatum*, and *C.spelaeum*.

## Supplementary Material

XML Treatment for Cynoglossum alticola

XML Treatment for Cynoglossum amabile

XML Treatment for Cynoglossum austroafricanum

XML Treatment for Cynoglossum coeruleum subsp. johnstonii var. mannii

XML Treatment for Cynoglossum hispidum

XML Treatment for Cynoglossum lanceolatum

XML Treatment for Cynoglossum obtusicalyx

XML Treatment for Cynoglossum spelaeum
